# Artificial intelligence and machine learning in the development of vaccines and immunotherapeutics—yesterday, today, and tomorrow

**DOI:** 10.3389/frai.2025.1620572

**Published:** 2025-07-18

**Authors:** Elhoucine Elfatimi, Yassir Lekbach, Swayam Prakash, Lbachir BenMohamed

**Affiliations:** ^1^Laboratory of Cellular and Molecular Immunology, College of Medicine, The Gavin Herbert Eye Institute, University of California, Irvine, Irvine, CA, United States; ^2^Institute for Immunology, University of California, Irvine Medical Center, Irvine, CA, United States; ^3^Chao Family Comprehensive Cancer Center, University of California, Irvine Medical Center, Irvine, CA, United States; ^4^Department of Vaccines and Immunotherapies, TechImmune, LLC, University Lab Partners, Irvine, CA, United States

**Keywords:** artificial intelligence (AI), machine learning (ML), vaccine development, infectious diseases, cancer, immunotherapeutics

## Abstract

The development of vaccines and immunotherapies against infectious diseases and cancers has been one of the significant achievements of medical science in the last century. Subunit vaccines offer key advantages over whole-inactivated or attenuated-pathogen-based vaccines, as they elicit more specific B-and T-cell responses with improved safety, immunogenicity, and protective efficacy. However, developing subunit vaccines is often cost-and time-consuming. In the past, the development of vaccines and immunotherapeutics relied heavily on trial-and-error experimentation, as well as extensive and costly *in vivo* testing, which typically required years of pre-clinical and clinical trials. Today, artificial intelligence (AI) and deep learning (DL) are actively transforming vaccine and immunotherapeutic research by (i) offering predictive frameworks that support rapid, data-driven decision-making, (ii) integrating computational models, systems vaccinology, and multi-omics data (iii) helping to better phenotype, differentiate, and classify patients diseases and cancers; (iv), integrating host characteristics for tailored vaccines and immunotherapeutics; (v) refining the selection of B-and T-cell antigen/epitope targets to enhance efficacy and durability of immune protection; and (vi) enabling a deeper understanding of immune regulation, immune evasion, and regulatory pathways. Artificial intelligence and DL are pushing the boundaries toward (i) the potential replacement of animal preclinical testing of vaccines and immunotherapeutics with computational-based models, as recently proposed by the United States NIH and FDA, and (ii) improving clinical trials by enabling real-time modeling for immune-bridging, predicting patients’ immune responses, safety, and protective efficacy to vaccines and immunotherapeutics. In this review, we describe the past and current applications of AI and DL as time-and resource-efficient strategies and discuss future challenges in implementing AI and DL as new transformative fields that may facilitate the rapid development of precision and personalized vaccines and immunotherapeutics for infectious diseases and cancers.

## 1 Introduction

Rapidly advancing immunology research has significantly contributed to the development of vaccines and immunotherapies against infectious diseases and cancers, marking a notable success in medical science. The relationship between artificial intelligence (AI) and immunology is intricate and transformative (Schultze et al., 2022). AI models are increasingly used to enhance our understanding of the cellular and molecular components of the immune system, leveraging computational power to identify complex interactions across immune pathways and disease contexts (Ekins et al., 2019; Topol, 2019; Vamathevan et al., 2019). Yesterday, immunological research relied primarily on experimental trial and error and time-consuming laboratory assays. These traditional methods, though foundational, were limited in scope and scalability. In the early days of computational immunology, traditional mathematical models were the primary tools for simulating immune responses. These early models relied on simplified assumptions and static parameters, often failing to account for the inherent complexity and variability of immune interactions across diverse populations. Before the implementation of artificial intelligence, immune system modeling was limited by computational constraints, small datasets, and the absence of real-time adaptability. This ‘yesterday’ phase laid the groundwork for today’s AI-driven approaches, highlighting both the potential and the limitations of classical immunological simulations. AI-driven models offer valuable insights into immunotherapeutic clinical trials, enhancing their delivery and efficacy. However, AI approaches can sometimes be misleading when they fail to consider the full complexity of immunological interactions, particularly when focusing on isolated mechanisms without integrating broader immunological networks. When experimental findings challenge existing AI-driven predictions, computational scientists must refine their models to ensure they remain aligned with empirical immunology. While AI-driven hypothesis generation can be insightful, the practical implementation of this approach remains a challenge. Many AI models lack robustness in real-world applications due to oversimplifications, biases in training datasets, and an inability to capture the full scope of immune system variability (Li et al., 2023). Moreover, specific AI models struggle to provide reliable experimental validation pathways, resulting in limitations to their clinical applicability (Xu et al., 2021).

There is significant debate among immunologists regarding (i) the role of AI-driven models in immunology (referred to in this report as immuno-AI) and (ii) how AI can be effectively employed to understand complex non-linear immunological systems (Xu et al., 2021). Immunology is inherently dynamic, involving multi-scale biological interactions that require advanced computational approaches for effective modeling. Today, AI and machine learning (ML) are at the forefront of immunological research, enabling unprecedented capabilities in simulating the immune system, mapping epitopes, and designing immunotherapies. AI has been instrumental in studying immune responses to viral infections (e.g., HIV-1), cancer (e.g., chronic myeloid leukemia), and autoimmune disorders (e.g., Alzheimer’s disease). Additionally, AI models have been informed by diverse research findings across physiology, behavior, and immunology. For instance, (Janssen et al., 2005) demonstrated how behavioral factors such as diet and physical activity influence health outcomes in youth populations, highlighting the need for integrative datasets. (Sato et al., 2021) further supported localized physiological modeling through resistance training outcomes. (Singh et al., 2023) emphasized the importance of physical activity in improving mental health, reinforcing the value of comprehensive models that encompass both psychological and physical health. From a cellular and mechanistic perspective, (Topchyan et al., 2023) provided evidence that CD4^+^ T cell help is essential for CD8^+^ T cell function in chronic infection and cancer, justifying the use of AI to simulate complex immune cell interactions. Traditional vaccine development relies on labor-intensive antigen screening, which can result in suboptimal formulations. AI-driven approaches have emerged as powerful tools to streamline epitope selection by leveraging diverse datasets, including single-cell RNA sequencing, structural protein modeling, and immune response profiling (Azevedo et al., 2024). Recent studies have demonstrated the potential of AI-powered frameworks, such as generative models and deep learning (DL) techniques, in accelerating vaccine design by predicting immune responses and optimizing multi-epitope formulations (Dogra et al., 2023; Guedan et al., 2019).

**Figure 1:**
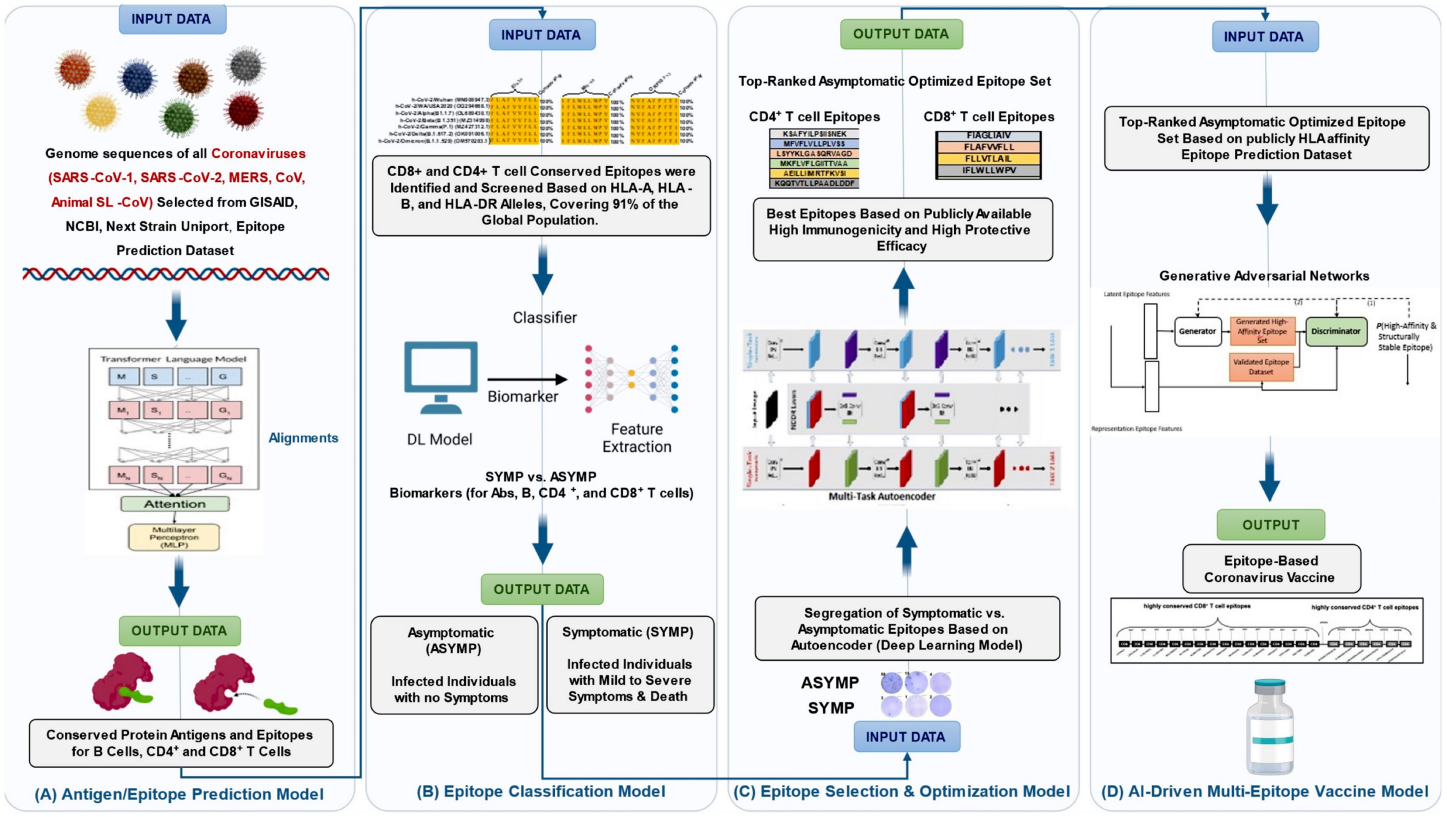
AI-driven framework optimizes b-and t-cell epitope prediction, classification, and multi-epitope vaccine design. **(A)** Epitope prediction model (transformer-based) for identifying immunogenic B-cell and T-cell epitopes, **(B)** deep learning epitope classification using (CNN-based) biomarker extraction and feature selection, **(C)** a multi-task autoencoder prioritizes immunogenic and protective epitopes, and **(D)** AI-powered vaccine formulation integrating top-ranked asymptomatic B-and T-cell epitopes into a candidate vaccine using Generative Adversarial Networks (GANs). GANs refine and generate four multi-epitope-based next-generation coronavirus vaccine candidates.

Artificial intelligence systems process large-scale immunological datasets, translating them into predictive frameworks that provide logical insights into immune responses, facilitate vaccine development, and guide novel immunotherapy strategies (Figure 1). Reflecting the growing confidence in AI-driven approaches, the U.S. FDA recently announced plans to phase out specific animal testing requirements and replace them with AI-based computational models and human organoid systems to enhance preclinical evaluation and improve translational relevance (Zushin et al., 2023). AI also excels in generating counterintuitive insights, uncovering immune interactions that may not be evident through conventional *in vitro* or *in vivo* experimental assays (Wherry and Kurachi, 2015). However, many immunologists remain skeptical about fully integrating AI-driven approaches, as computational models often differ from traditional wet-lab methodologies (Borst et al., 2018). The emerging field of immuno-AI aims to bridge this gap by fostering interdisciplinary collaboration between AI researchers and immunologists. AI models are playing a transformative role in vaccine and immunotherapy development by enabling more precise predictions of immune responses. Deep learning-based classifiers, such as convolutional neural networks (CNNs), have been utilized to differentiate protective versus non-protective immune responses (Zhou et al., 2010). By integrating AI-powered biomarker discovery, these models contribute to refining vaccine formulations that enhance and prolong immunity. AI-driven immunogenicity prediction frameworks, validated through experimental approaches, demonstrate their potential in optimizing vaccine efficacy and guiding next-generation immunotherapy strategies (Kaech and Cui, 2012).

Artificial intelligence plays a crucial role in deciphering the complex, non-linear dynamics of the immune system. Most immunological interactions involve complex feedback loops and multi-scale regulatory mechanisms. The immune system is highly non-linear, meaning that small perturbations in immune signals can lead to disproportionate effects on immune responses. A key example is T-cell activation in response to antigen (Ag) concentration (Pardoll, 2012). T cells exhibit threshold-based responses, where minimal antigen exposure triggers no response, while increased antigen concentration induces an exponential increase in activation. However, at extremely high antigen levels, T-cell responses may decline due to immune exhaustion mechanisms (e.g., PD1^+^CD8^+^ or TIM^+^CD8^+^ T cells) (Schapiro et al., 2022). While immunologists have long studied dose–response relationships, AI enables a more detailed exploration of how immune responses dynamically shift across different conditions. The complexity of cytokine networks, chemokine interactions, and immune checkpoints makes AI-based models particularly valuable for predicting immune system behaviors in health and disease (van Dorp et al., 2025).

One of the most significant challenges AI researchers face in immunology is capturing the full complexity of immune phenomena. Immunologists themselves often struggle to fully decipher immunological mechanisms due to current limitations in experimental assays and data interpretation. AI models must address critical gaps in immune system modeling, including predicting the speed of immune responses, analyzing T-cell subset differentiation (Th1, Th2, Th17), and simulating immune activation under diverse conditions (Kaech and Cui, 2012). AI has been applied to modeling CD4^+^ T cell differentiation, but many studies remain theoretical and lack robust experimental validation (Lu et al., 2021). To overcome these challenges, AI-driven models must strike a balance between complexity and interpretability. Both immunologists and AI scientists often rely on simplified models that capture essential immune dynamics without excessive abstraction. AI enables pattern recognition, predictive analytics, and experimental design improvements, allowing researchers to identify key immune parameters for hypothesis testing (Wang et al., 2023). However, over-reliance on AI without validation through empirical immunology can lead to misleading conclusions. Base immune-like AI models should be viewed as complementary to wet-lab immunology rather than as standalone predictive tools. Advances in user-friendly computational tools have facilitated the rapid expansion of AI applications in immunology. These platforms enable immunologists to integrate AI-driven insights into their research without requiring extensive programming expertise. AI models often begin with fundamental immunological assumptions, which are refined through iterative machine-learning approaches. However, AI-driven predictions must be carefully scrutinized to ensure their applicability to real-world immunological settings. While AI models have led to numerous breakthroughs, such as AstraZeneca’s use of AI to inform cancer drug trials, challenges persist in translating computational insights into clinically actionable immunological strategies.

A growing number of AI approaches aim to simulate adaptive immune responses, including the activation of B cells and T cells (Miho et al., 2018; Isacchini et al., 2021). AI-based immune simulations can generate realistic predictions that align with experimental findings, but many models remain overly simplified. One limitation is the failure to account for the dynamic interplay between multiple immune components (Bottcher et al., 2023). AI simulations should prioritize mechanistic insights that can be experimentally validated rather than focusing solely on predictive accuracy. Immunologists often express concerns about AI models omitting critical immune variables. However, just as experimental studies focus on a subset of parameters (e.g., antigen load, cytokine levels, and immune cell counts), AI-driven approaches can provide valuable insights by identifying essential immune interactions (Bottcher et al., 2023; Thomas et al., 2022). Simple AI models can be beneficial for ruling out ineffective immune mechanisms rather than solely predicting successful outcomes. AI-based models are also increasingly employed in vaccine design and immunotherapy development (Zhao et al., 2024; Olawade et al., 2024; Ong et al., 2020b). By optimizing clinical trial parameters, AI can reduce the number of experimental groups, refine vaccine formulations, and optimize immune checkpoint inhibitor protocols (Olawade et al., 2024). Recent studies continue to demonstrate the expanding role of AI in epidemiological and immunological modeling. For example, Çolak developed a multilayer perceptron-based artificial neural network to simulate and predict COVID-19 infection and mortality rates in Turkey, demonstrating high predictive performance and highlighting the value of AI in pandemic forecasting and response planning (Çolak, 2021). Similarly, Shafiq et al. conducted a comparative analysis between artificial neural networks (ANN) and parametric models for COVID-19 data prediction, demonstrating that ANN-based models yielded superior forecasting accuracy and robustness for epidemiological analysis (Shafiq et al., 2022). These studies highlight the urgent need for adaptable and interpretable AI tools in infectious disease research. AI can guide vaccine development by predicting immunogenic epitopes, assessing immune memory responses, and evaluating vaccine efficacy across diverse populations (Paul et al., 2013; Villani et al., 2018). Tomorrow, the integration of advanced AI frameworks, including generative models, multi-modal learning, and interpretable machine learning, will further accelerate the design of personalized vaccines and immunotherapies, creating a future where immune responses can be simulated and optimized in silico before entering clinical pipelines.

In summary, AI models are transforming immunological research by facilitating experimental design, identifying key immune interactions, and optimizing therapeutic strategies (Schubert, 2011; Gasperini et al., 2025). AI enables the rapid generation of hypotheses and computational simulations that guide experimental immunology in new directions (Gao et al., 2024). Under a broader “systems immunology” perspective, AI helps immunologists (i) to focus investigations for rapid discovery and (ii) to identify immune mechanisms that may not work as expected (Miho et al., 2018; Gasperini et al., 2025). The integration of AI with wet lab immunology is crucial for advancing the field, fostering interdisciplinary collaboration, and ensuring AI-driven insights contribute to real-world immunological advancements (Flower and Doytchinova, 2002). The following sections will explore the complexities of immunological systems and emphasize the necessity of improved collaboration between AI researchers and immunologists (Davis et al., 2017).

This work is novel in its dual purpose: it provides a comprehensive review of the evolving role of artificial intelligence in vaccines and immunotherapeutics while also including a practical transformer-based deep learning case study. The case study exemplifies how cutting-edge AI can be directly applied to immunological problems, addressing a key gap in the literature where theoretical discussions often lack real-world modeling demonstrations. By combining critical review with practical implementation, our work bridges the disconnect between AI theory and translational application in immunology and vaccinology.

### 1.1 AI-driven modeling and nonlinear dynamic immune systems

The non-linearity of biological systems presents significant challenges in understanding immune responses, necessitating the use of artificial intelligence (AI)-driven modeling to complement experimental approaches (Callard and Yates, 2005). Most immunological processes involve nonlinear interactions between cellular and molecular components, often incorporating both positive and negative feedback loops (Feinerman et al., 2010). Because immune responses do not always exhibit proportional input–output relationships, AI-based computational models are essential for capturing the complexity of these interactions and predicting immune system behaviors (Miao et al., 2010). For instance, AI-based simulations of T-cell responses have demonstrated that immune activation is highly dependent on antigen (Ag) concentration (Wu et al., 2011). Supplementary Figure S3 illustrates the predicted T cell proliferation response to varying antigen concentrations, modeled using a saturating function. The model assumes that the T cell proliferation rate depends on antigen concentration according to a function (Barron et al., 1994; Lairson et al., 2003; Pepose et al., 2006; Sykakis et al., 2013).

$$\frac{\mathrm{\rho I}}{\left(h+I\right)}$$

Where ρ represents the maximum proliferation rate, I is the antigen concentration, and h is the half-saturation constant. We assume that immune response starts with 100 T cells, ρ = 1 $\;{\mathrm{day}}^{-1}$ , and T cells divide for 1 week.

AI-driven modeling provides valuable insights into nonlinear immune dynamics, particularly in T-cell proliferation in response to antigen concentration (Ganusov et al., 2011). Supplementary Figure S3 demonstrates how T cell expansion follows a saturating function, indicating that immune activation is highly dependent on antigen availability. The sigmoidal response curves illustrate that as antigen concentration increases, T cell proliferation initially follows a slow growth phase, then enters a rapid expansion phase before reaching a plateau (Toussaint et al., 2008). The shape of these curves is governed by the half-saturation constant (h), which determines the antigen threshold required to trigger significant T-cell proliferation. A lower h value (h = 0.01, blue curve) results in an earlier and steeper immune response, meaning that T cells respond efficiently to trim antigen levels.

In contrast, a higher h value (h = 0.1, red curve) shifts the activation threshold, requiring a higher antigen concentration to induce the same level of immune response (Terry and Chaplain, 2011). These findings underscore the importance of optimizing antigen dose in immunotherapy and vaccine design (Pappalardo et al., 2010). Traditional mathematical models, while helpful in describing basic immune activation principles, often fail to account for real-world immune variability (Davenport et al., 2004). AI-driven models enhance these predictions by integrating multi-omics datasets, real-time patient profiling, and adaptive learning algorithms to dynamically refine T cell activation thresholds (Callard and Yates, 2005; Barron et al., 1994; Lairson et al., 2003; Pepose et al., 2006; Sykakis et al., 2013). This results in more accurate predictions of immune memory formation, therapeutic efficacy, and antigen dose–response relationships, ultimately enhancing the development of personalized immunotherapies and precision vaccines (Feinerman et al., 2010).

**Figure 2:**
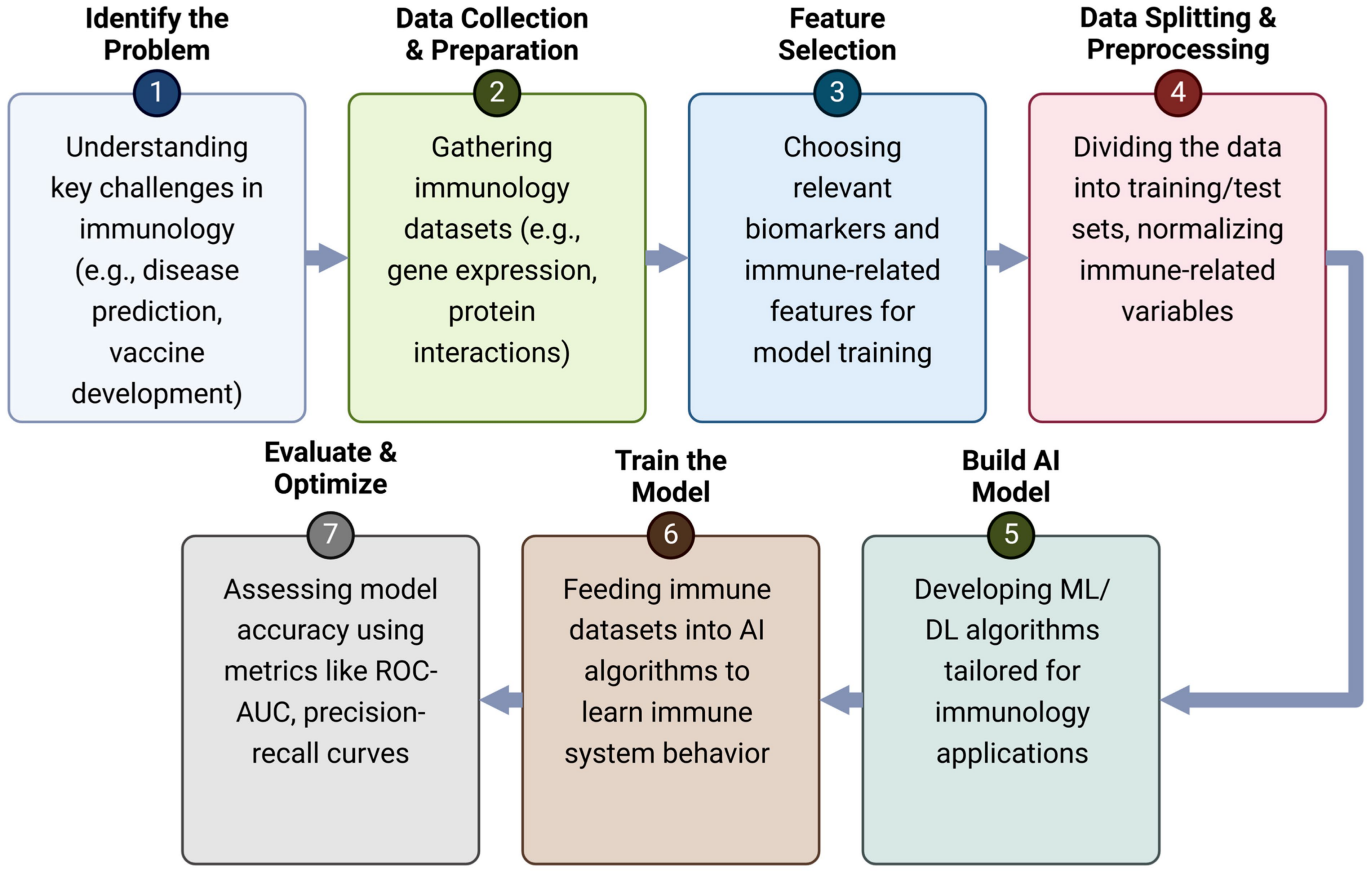
AI-driven workflow for immunology research. This illustration presents a structured framework outlining the role of Artificial Intelligence (AI) in immunology research, demonstrating how AI-driven approaches enhance immunological analysis, predictive modeling, and therapeutic development. The process begins with the identification of key immunological challenges, such as disease prediction and vaccine development, followed by the collection and preprocessing of immunological datasets, including gene expression profiles and protein interactions. Feature selection then plays a crucial role in identifying relevant biomarkers and immune-related variables to improve model accuracy. Once the data is prepared, machine learning and deep learning models are built and trained on immune system datasets to recognize patterns and predict immune responses. The trained models undergo evaluation and optimization using performance metrics like ROC-AUC and precision-recall curves to ensure accuracy and reliability. Ultimately, these AI-driven models are applied in real-world immunology research, enabling advancements in personalized medicine, immunotherapy, and vaccine optimization. By integrating AI techniques into immunology, researchers can gain deeper insights into immune system behavior, develop more effective therapeutic interventions, and refine disease treatment strategies.

Artificial intelligence models have shown that T cells do not activate until Ag levels cross a specific threshold, after which there is an exponential increase in response magnitude before reaching a plateau (Wu et al., 2011; Barron et al., 1994; Lairson et al., 2003; Pepose et al., 2006). At excessively high Ag levels, T cell exhaustion can occur, characterized by the upregulation of immune checkpoints such as PD1^+^CD8^+^ or TIM^+^CD8^+^ T cells, leading to immune suppression and decreased functionality (Wu et al., 2011). Traditional dose–response models often fail to fully capture these dynamics. In contrast, AI-based predictive frameworks can adapt and refine immunological interpretations in real-time, leading to more accurate therapeutic interventions (Cheng et al., 2009). AI-driven models are particularly advantageous in predicting the behavior of cytokine and chemokine networks (Ganusov et al., 2011). While traditional approaches often struggle to model complex immune signaling pathways, AI algorithms such as deep learning and reinforcement learning models enable real-time adjustments based on experimental immunological data (Toussaint et al., 2008). The structured workflow of artificial intelligence (AI) in immunology research is shown in Figure 2. The process begins with identifying key immunological challenges and progresses through data collection, feature selection, and data preprocessing. AI models are then developed and trained using immune datasets, followed by evaluation and optimization through performance metrics such as ROC-AUC and precision-recall curves. Finally, the AI-driven models are deployed for clinical testing and real-world application in immunology, vaccine development, and immunotherapy. This workflow showcases the integration of AI techniques to enhance immunological research, improve predictive modeling, and optimize therapeutic strategies, ensuring that AI-driven models can efficiently analyze immune system dynamics and inform the development of vaccines and immunotherapy strategies.

**Figure 3:**
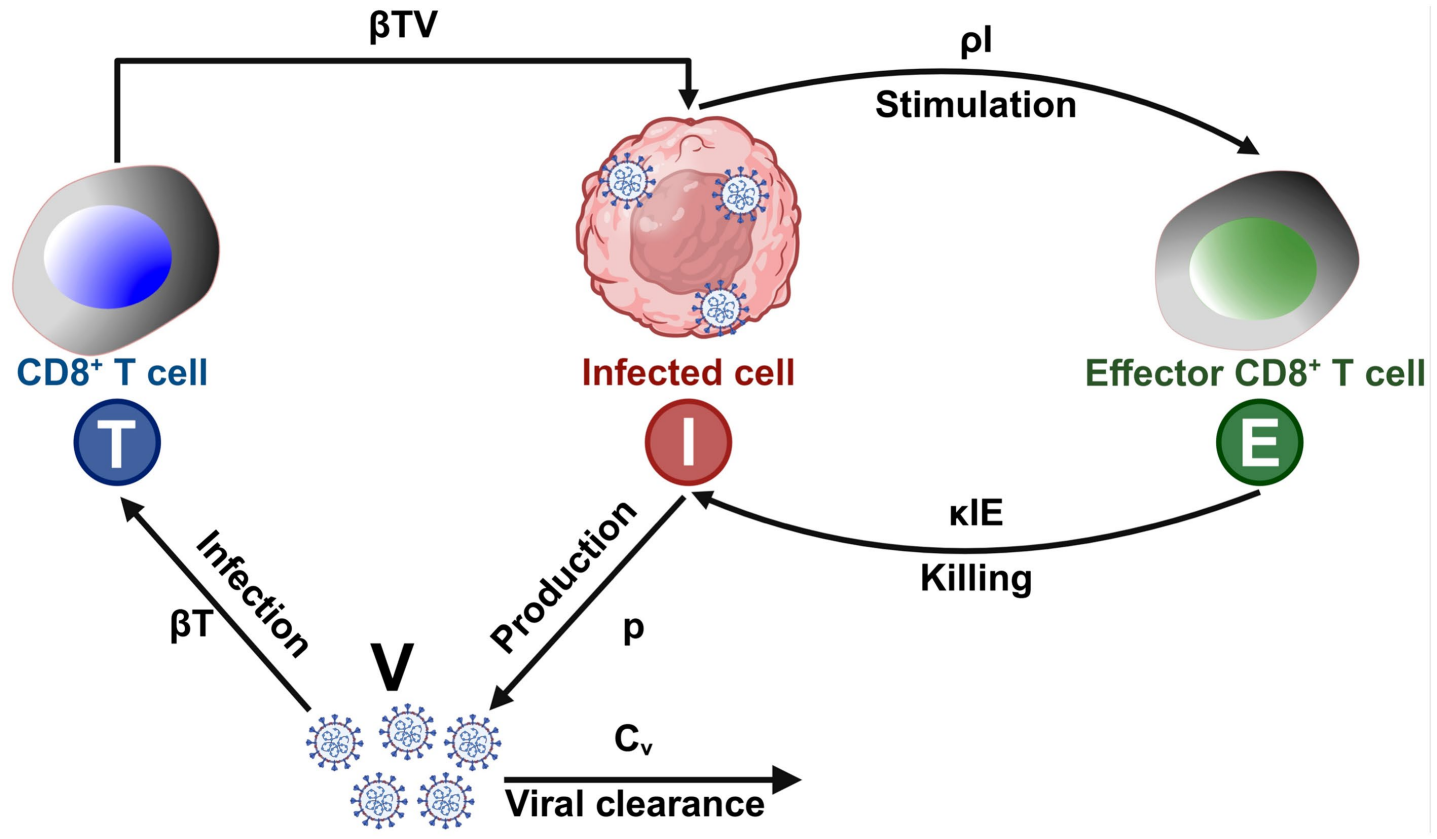
AI-driven mathematical model of CD8^+^ T cell response to viral infections: T (Blue) – CD8^+^ T cells that recognize and eliminate infected cells; I (Red) – Virus-infected cells that replicate and produce viral particles; E (Green) – Effector immune cells (e.g., activated CD8^+^ T cells); V – Free virus particles that infect host cells; βT – Rate at which virus infects host cells and generates infected cells (I); βTV – Interaction coefficient representing how CD8^+^ T cells (T) are stimulated by viral load (V); p – Rate of virus production by infected cells (I); κIE – Killing rate of infected cells (I) by effector T cells (E); ρI – Stimulation coefficient, representing the activation of effector immune cells (E) by infected cells (I); Cv – Clearance rate of virus (V) due to immune response.

The ability of AI-driven models to efficiently analyze immune system dynamics is particularly valuable when studying complex immunological processes such as CD8^+^ T-cell responses to antigenic peptides (Wherry, 2011). For example, the unpredictable CD8^+^ T-cell response in IL-2^−/−^ or IL-2R^−/−^ knockout mice has historically been attributed to redundancy within the immune system, with IL-15 compensating for the loss of IL-2 function (Miho et al., 2018). However, AI models provide a more nuanced perspective, demonstrating that cytokine networks maintain stability through compensatory mechanisms rather than simple redundancy (Pandya et al., 2021). These insights are crucial for refining immune intervention strategies and designing more effective immunotherapies (Chentoufi et al., 2011; Coulon et al., 2024; Prakash et al., 2024b; Prakash et al., 2024a; Zayou et al., 2025; Prakash et al., 2021). The immune system is an extraordinarily complex network of interacting cells and molecules that function collectively to mediate immune-protective, immune-pathologic, or immune-evasion responses against infectious pathogens or cancers (Ganusov et al., 2011). This includes, for example, the induction of cytotoxic CD8^+^ T cells that eliminate HIV-infected target cells or inhibit the proliferation of tumor cells in breast cancer (Toussaint et al., 2008). AI-based modeling has proven to be invaluable in identifying key regulatory interactions governing immune responses, including cytokine and chemokine signaling at infection or tumor sites, antigen-presenting cell (APC) activation and migration, antigen presentation to T cells, and CD4^+^/CD8^+^ T-cell cooperation with dendritic cells in infected or tumor tissues and lymph nodes (Terry and Chaplain, 2011). We have presented a mathematical model of the CD8^+^ T cell response to viral infections, highlighting the interplay of positive and negative feedback loops in immune regulation (Figure 3). AI-driven models enhance traditional frameworks by dynamically adapting to experimental data and refining predictions regarding T cell activation, viral replication, and immune clearance. Unlike static models, AI-based simulations integrate real-time immune data, identifying patterns of viral persistence, immune exhaustion, and immune evasion that were previously difficult to capture (Chentoufi et al., 2011; Coulon et al., 2024; Prakash et al., 2024b; Prakash et al., 2024a; Zayou et al., 2025; Prakash et al., 2021). By leveraging machine learning algorithms, AI enhances our understanding of immune checkpoints, cytokine signaling, and antigen presentation, allowing for the refinement of predictive models for disease progression and therapeutic interventions (Davenport et al., 2004).

Despite advancements in AI-driven immunology, challenges persist in bridging experimental data with computational modeling. Many immunologists struggle to fully grasp the complexity of AI-driven immune simulations, mainly due to the limitations of current immunological assays and the uncertainty in interpreting observed phenomena (Callard and Yates, 2005). AI frameworks must continuously improve by integrating multi-omics datasets, refining immune system simulations, and enhancing data-driven decision-making processes (Barron et al., 1994; Lairson et al., 2003; Pepose et al., 2006; Sykakis et al., 2013). The development of user-friendly AI-driven software tools in the past two decades has empowered immunologists to explore computational models with greater accessibility. These tools facilitate real-time data integration, enabling researchers to test hypotheses through predictive immune modeling. However, one major challenge is that many AI-driven models rely on predefined assumptions and parameter tuning that may not accurately reflect the real-world behaviors of the immune system. This has led to an increasing body of work where AI-generated predictions have been overinterpreted, sometimes without direct experimental validation.

AI-based simulations have also been employed to study B-cell and T-cell responses, offering a more biologically relevant representation of adaptive immunity compared to static models (Miho et al., 2018). Unlike traditional mathematical frameworks that often omit key immunological interactions, AI-driven immunology incorporates large-scale immune cell interactions, antigen processing, and host-pathogen dynamics, ensuring a more realistic and holistic understanding of immune responses. AI models are now advancing to simulate multi-dimensional immune responses, including the simultaneous calculation of speeds for multiple immune pathways, the dynamics of T-cell proliferation, and interaction modeling between Th1, Th2, and Th17 responses. Unlike previous models, AI enables real-time tracking of antigen presentation, immune exhaustion dynamics, and cross-reactivity in vaccine design. While immunologists often express concern when AI models do not incorporate all known variables of the immune system, these models can still be invaluable for hypothesis testing (Eftimie et al., 2016). AI-driven frameworks can identify immune pathways that are unlikely to be effective, allowing researchers to focus experimental efforts on the most promising targets. In clinical applications, AI models have proven beneficial in optimizing vaccine development, reducing the number of experimental groups, refining immunization schedules, and predicting long-term immune memory formation (Greiff et al., 2015). AI-driven models are revolutionizing immunology by offering predictive power at every stage of immune research, from experimental design to therapeutic development (Pulendran and Davis, 2020). Unlike traditional computational approaches, AI integrates multi-dimensional immune data to identify key regulatory mechanisms, predict immune outcomes, and refine treatment strategies (Villani et al., 2018). AI enhances immunological research by providing real-time predictive insights into immune dynamics, enabling rapid adjustments to experimental design and protocols. This allows the identification of key immune interactions that drive disease progression, leading to more targeted immunotherapeutic interventions.

Additionally, AI improves vaccine and drug development by allowing data-driven optimization of immunization strategies and treatment protocols. To maximize the potential of AI in immunology, interdisciplinary collaboration is critical; AI scientists, immunologists, and clinicians must work together to ensure that AI-driven predictions align with real-world immunological mechanisms (Wouters et al., 2020). By advancing AI-based immune modeling, the scientific community can accelerate breakthroughs in precision medicine, immunotherapy, and vaccine development, ultimately leading to improved patient outcomes and disease management (Gao et al., 2024).

### 1.2 AI-driven modeling for phenotype, differentiation, and classification of diseases and cancers

Artificial intelligence (AI) is revolutionizing the fields of disease phenotyping, patient stratification, and cancer classification, driving a paradigm shift toward precision medicine. AI models, particularly those based on advanced architectures such as convolutional neural networks (CNNs), graph neural networks (GNNs), generative adversarial neural networks (GAN), and transformer-based approaches, have demonstrated an unprecedented capacity to integrate and analyze complex multi-modal datasets, including genomics, transcriptomics, proteomics, radiomics, and clinical records (Li et al., 2024; Garg et al., 2024). In the context of neurodegenerative diseases, such as Alzheimer’s disease (AD) and mild cognitive impairment (MCI), AI-driven systems have enhanced diagnostic accuracy by distinguishing subtle differences in cognitive decline trajectories that are often imperceptible to human evaluators (Tascedda et al., 2024; Borchert et al., 2023). Recent studies employing GNNs, and deep learning models have successfully mapped patient similarities based on cognitive, genetic, and neuroimaging features, achieving higher predictive power in differentiating between AD, MCI, and healthy controls compared to conventional methods (Tascedda et al., 2024). In oncology, AI algorithms have enabled the classification of tumors by extracting hidden patterns from histopathological images and molecular signatures, facilitating the identification of prognostic biomarkers and informing therapeutic decision-making. These AI-guided stratification models not only replicate existing diagnostic pathways but also uncover novel disease subtypes and phenotypic variations that were previously unrecognized, thereby opening new avenues for targeted therapy development. Notably, by learning directly from large, heterogeneous patient datasets, AI systems are mitigating the biases inherent in traditional clinical decision-making and enabling a more individualized and equitable approach to healthcare delivery. Collectively, the integration of AI into disease differentiation and classification workflows represents a transformative advancement in the biomedical sciences, enabling earlier detection, improved prognostication, and personalized interventions that are poised to improve patient outcomes across a spectrum of complex diseases significantly.

#### 1.2.1 AI-driven modeling for vaccine and immunotherapy design

**Figure 4:**
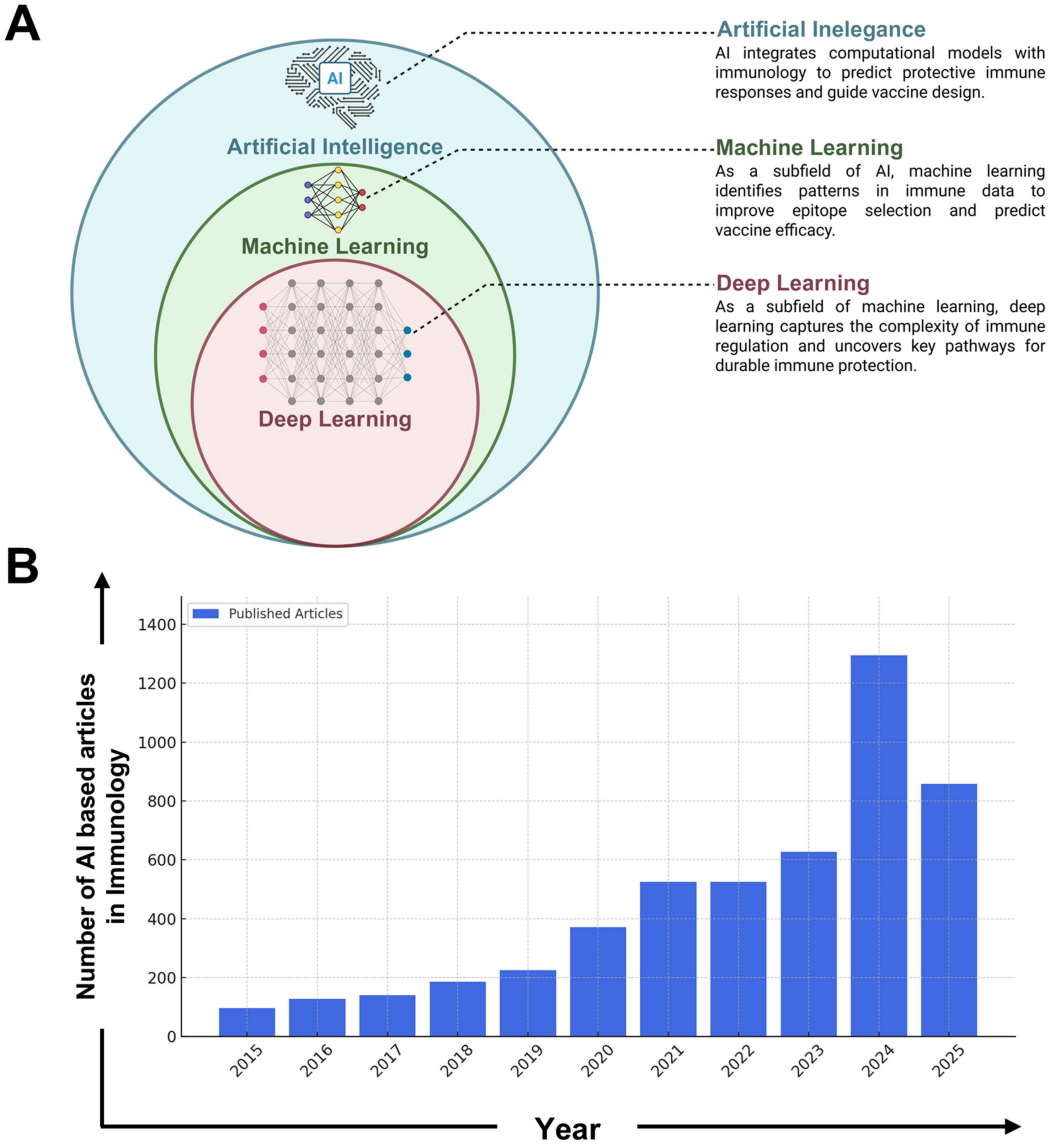
Growth of AI-driven research in immunology (2015–2024). **(A)** Conceptual representation of the hierarchical relationship among Artificial Intelligence (AI), Machine Learning (ML), and Deep Learning (DL) in the context of immunological applications. AI serves as the broadest category, encompassing ML techniques that identify patterns in immune-related datasets. DL, a specialized subfield of ML, is used to model complex immune responses and uncover pathways critical for vaccine efficacy and immune regulation. **(B)** The integration of Artificial Intelligence (AI) in immunology has undergone significant expansion over the past decade. The number of publications in this field has grown from just over 100 articles per year in 2015 to over 1,500 in 2024, highlighting the increasing role of AI in immunological research and its applications in vaccine design, immune response modeling, and precision medicine. This diagram was generated based on keyword searches such as “AI in immunology,” “machine learning in immunology,” and “deep learning in immune research” from the PubMed database. The search encompassed peer-reviewed articles, systematic reviews, and conference proceedings related to AI-driven immunological studies. The data reflects the rising trend in AI applications, including areas such as predictive immune modeling, AI-assisted diagnostics, and computational vaccine development. The exponential rise in publications post-2020 aligns with breakthroughs in deep learning, large-scale immunological datasets, and AI-driven drug discovery, indicating a paradigm shift in how computational tools are being utilized in immunology. The projected increase in AI-based studies suggests continued advancements in immune system modeling, personalized immunotherapy, and AI-enhanced vaccine development strategies.

Artificial intelligence (AI)-driven modeling has been extensively applied to optimize and enhance immunotherapeutic vaccine strategies against infectious pathogens and cancers while also evaluating their efficacy, as illustrated in Figures 4A,B. As illustrated in Figure 4A, the hierarchical structure of AI, encompassing machine learning (ML) and deep learning (DL), highlights their distinct roles in immunological research. AI serves as the overarching framework, integrating computational models to predict immune responses and support the design of vaccines. Machine learning (ML) methods are used to identify patterns in immune-related data, while deep learning (DL) techniques model complex immune interactions and uncover critical regulatory pathways. Together, these approaches provide a robust foundation for data-driven vaccine development. AI models are used to predict the magnitude of immune responses across various immune cell populations *in vitro*. Due to the inherent uncertainty in predicting the nature of immune responses elicited by a given vaccine, multiple AI-driven models are applied. Results from subsequent experimental testing help to validate AI models, allowing for the rejection of inaccurate assumptions while refining those that best align with observed immune responses. This iterative approach enhances the understanding of immune mechanisms underlying vaccine efficacy. AI-validated models are then utilized to optimize vaccine delivery strategies, thereby enhancing immunization effectiveness and reducing the need for unnecessary experimental trials. Compared to conventional drug development, vaccine development is a lengthy and costly process that requires extensive laboratory experiments to assess safety, immunogenicity, and efficacy. Compared to traditional vaccine development, which relies on trial-and-error antigen screening, AI-driven methods leverage predictive modeling to optimize vaccine formulation. AI algorithms integrate multi-omics datasets, simulate immune responses, and identify the most promising epitope candidates for immunization. This computational approach reduces vaccine development time, lowers costs, and enhances success rates by filtering out ineffective candidates before clinical trials.

AI-driven modeling substantially reduces costs by eliminating redundant experiments and narrowing down vaccine candidates before clinical trials. As immunological efficacy is demonstrated through preclinical and clinical trials, one major challenge remains: determining the optimal vaccine dosage. AI-based approaches systematically explore optimal dosing strategies and vaccination schedules, predicting reductions in vaccine injections by approximately 30% compared to traditional *in vivo* experiments. The comparison between conventional and AI-driven immunotherapy development highlights the significant improvements AI brings to drug discovery. Traditional immunotherapy approaches typically take around 6 years (72 months) and cost an estimated $500 million per drug, with a low success rate of approximately 10%, as many drug candidates fail to progress beyond early trial stages (Wouters et al., 2020).

In contrast, AI-driven methods leverage deep learning and predictive modeling to reduce development time to about 2 years (24 months) and lower costs to around $150 million per drug while increasing the success rate to approximately 30% (Wouters et al., 2020). AI achieves these improvements by optimizing the selection of drug candidates, identifying potential failures earlier, and accelerating the overall research pipeline. Studies report that AI-driven approaches significantly enhance the efficiency of early-stage drug discovery, reducing costs by up to 70–80% while improving screening accuracy (Ekins et al., 2019). These findings demonstrate the transformative impact of AI in immunotherapy, offering faster, more cost-effective, and higher-success drug development pathways (Lo et al., 2014).

A critical component of AI-driven vaccine development is the integration of specialized AI models that address key aspects of immune response prediction and vaccine formulation. These models ensure that vaccine candidates elicit broad and durable immune protection. The Antigen/Epitope Prediction Model (Figure 1A) utilizes transformer-based deep learning to identify conserved B-and T-cell epitopes across multiple viral variants, integrating genomic, structural, and immunological datasets to optimize vaccine targets. The Epitope Classification Model (Figure 1B) utilizes convolutional neural networks (CNNs) to classify protective versus non-protective immune responses based on symptomatic and asymptomatic patient datasets, thereby refining epitope selection for enhanced immunogenicity. The Epitope Selection & Optimization Model (Figure 1C) incorporates a multi-task autoencoder to prioritize epitopes that exhibit high immunogenic potential while minimizing immune escape risks. This model integrates HLA-affinity screening, single-cell RNA sequencing, and interaction probability maps to enhance vaccine design and development. The AI-driven multi-epitope Vaccine Model (Figure 1D) employs generative adversarial networks (GANs) to refine multi-epitope vaccine formulations, ensuring the inclusion of high-affinity epitopes optimized for antigen presentation and immune activation.

**Figure 5:**
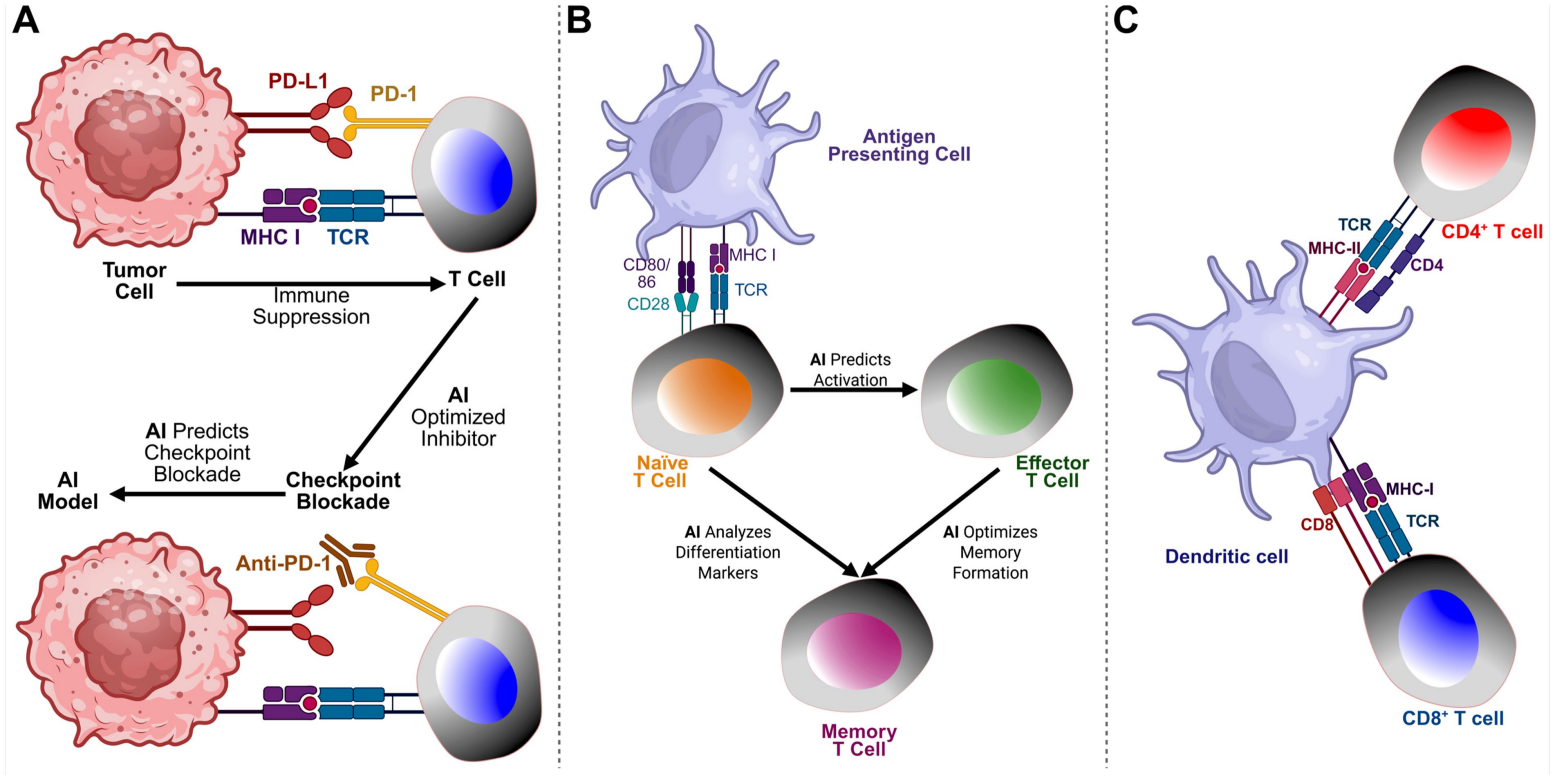
AI-driven insights into tumor-immune interactions and memory T cell differentiation. **(A)** AI’s Role in Tumor-Immune Interactions and Checkpoint Blockade: This panel illustrates how Artificial Intelligence (AI) supports understanding and optimization of tumor-immune interactions, particularly in regulating immune checkpoint inhibitors. Tumor cells suppress T cell activity through checkpoint pathways such as PD-1/PD-L1 and CTLA-4. AI models predict optimal checkpoint blockade strategies by identifying patient-specific responses, discovering biomarkers, and enhancing the efficacy of combination immunotherapies. Integration of multi-omics data (genomics, transcriptomics, proteomics) via AI enables personalized immunotherapy with improved efficacy and reduced toxicity. **(B)** AI Insights into Memory T-Cell Differentiation: This panel illustrates the role of AI in guiding the differentiation of memory T cells. Following antigen exposure, naïve T cells activate and differentiate into effector and memory T cells. AI enhances this process by predicting activation based on TCR signaling, analyzing differentiation markers, and optimizing memory formation. AI models trained on single-cell RNA sequencing and epigenetic data uncover pathways critical for long-term immune protection, informing vaccine development and cancer immunotherapies. **(C)** Interaction Between Dendritic Cells and T Cells in Antigen Presentation. This illustration depicts the crucial role of dendritic cells (DCs) in bridging innate and adaptive immunity through antigen presentation. The dendritic cell presents antigens to CD4^+^ T cells via MHC class II molecules, which are recognized by the T cell receptor (TCR) in conjunction with the CD4 co-receptor, facilitating the activation of helper T cells. Simultaneously, CD8^+^ T cells recognize antigens presented on MHC class I molecules, with the TCR engaging the complex alongside the CD8 co-receptor, resulting in the activation of cytotoxic T cells. This dual interaction is essential for initiating and coordinating immune responses, enabling helper T cells to support other immune cells and cytotoxic T cells to eliminate infected or malignant cells.

Beyond epitope selection, AI-driven models also predict vaccine durability by analyzing immune exhaustion and antigenic persistence. AI models have demonstrated that prolonged antigen exposure without adequate control can lead to T cell exhaustion, characterized by the upregulation of inhibitory markers such as PD-1 and TIM-3, which ultimately impairs vaccine-induced immunity (Wu et al., 2011). This is particularly relevant for vaccines targeting chronic infections, such as herpesvirus-based vaccines, where AI-driven approaches underscore the need for targeted immune stimulation at viral reactivation sites, including the trigeminal and sacral ganglia. AI-driven models suggest that optimizing localized immune responses at these sites enhances protective immunity, surpassing the efficacy of systemic immune activation alone. The role of AI in peptide-based vaccine development has also been extensively studied. AI models assist in designing peptide-based CD8^+^ T cell vaccines against HSV, HIV-1, SARS-CoV-2, and malaria, predicting optimal short peptide epitopes that exhibit high binding affinity to MHC class I molecules (Ganusov et al., 2011; Chentoufi et al., 2011; Prakash et al., 2024b). Compared to whole-protein vaccines, epitope-based vaccines offer greater immunogenic precision, enabling the inclusion of multiple immunodominant and subdominant epitopes within a single antigenic formulation. However, one of the significant challenges in epitope-based vaccine design is the high degree of HLA polymorphism, which could limit broad population coverage. AI-driven modeling addresses this limitation by incorporating supertype-restricted epitopes recognized by diverse HLA alleles. For example, AI models predict that a Th-CTL peptide-based herpes vaccine should include multiple CD8^+^ T cell epitopes derived from herpesvirus proteins, designed to cover HLA-A2, HLA-A3, and HLA-B7 supertypes, which collectively ensure immune recognition in a large portion of the global population (Barron et al., 1994; Lairson et al., 2003; Pepose et al., 2006; Sykakis et al., 2013). By leveraging AI-driven epitope mapping, researchers can identify HLA class I-degenerate T cell epitopes, facilitating the development of multi-epitope Th-CTL peptide vaccines with broad immunogenicity (Figure 5A).

The selected CD8^+^ T cell epitopes target HLA-A2, HLA-A3, and HLA-B7 supertypes, which are among the most prevalent across global populations. For example, the HLA-A*02:01 allele (within the A2 supertype) occurs in approximately 20–50% of individuals in European, East Asian, and Latin American populations (Solberg et al., 2008). Similarly, HLA-A3 and HLA-B7 supertypes are moderately prevalent in many populations worldwide (Sidney et al., 2008). Together, these supertypes fall within a group of nine primary HLA class I supertypes (A1, A2, A3, A24, B7, B27, B44, B58, and B62) that collectively cover over 95% of the global population in terms of epitope-binding potential (Greenbaum et al., 2011). This high cumulative population coverage underscores the strategic value of selecting epitopes that bind to A2, A3, and B7 supertypes for the design of a broadly protective vaccine.

AI-driven modeling has also proven instrumental in understanding co-infections and their impact on immune responses. Many individuals harbor multiple pathogens simultaneously, leading to both positive and negative immuno-synergies between infections. AI-based models have been applied to study the dynamics of co-infections, optimizing vaccine formulations for individuals affected by multiple pathogens. For instance, AI-driven models addressing HSV-HIV co-infections provide valuable insights into immune evasion mechanisms and highlight novel immunotherapeutic targets that traditional approaches may overlook. These findings have broad implications for vaccine design strategies, particularly in immunocompromised populations. By integrating AI into vaccine development, researchers can enhance vaccine efficacy, refine immunization schedules, and minimize immune escape mechanisms. AI-driven approaches ensure that vaccine candidates undergo rigorous computational and experimental validation, allowing for faster, more effective, and scalable vaccine development to combat emerging infectious diseases. As AI technologies continue to advance, their integration with immunology is expected to play a crucial role in the development of next-generation, personalized, and precision-based vaccines.

##### 1.2.1.1 Use of artificial intelligence in the development of vaccines and immunotherapeutics for infectious diseases

**Figure 6:**
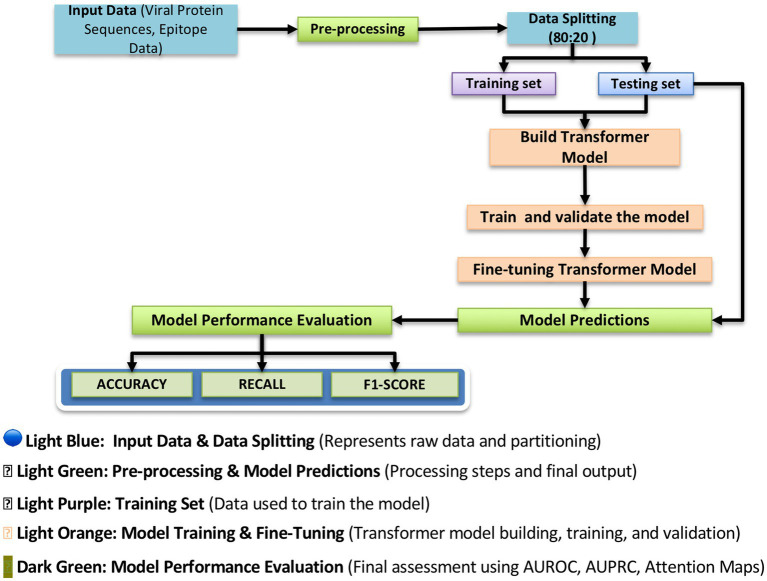
Antigen/epitope prediction model workflow. This figure illustrates the structured workflow of the transformer-based deep learning model used for antigen and epitope prediction. The pipeline begins with input data, consisting of viral protein sequences and epitope data, which undergo preprocessing to extract relevant features. The dataset is then split into training (80%) and testing (20%) subsets to ensure proper model generalization. The transformer-based model is trained and fine-tuned using deep learning techniques to improve prediction accuracy. Finally, the model undergoes performance evaluation using metrics such as Accuracy, Recall, and F1-score, providing insight into the reliability of predicted immunogenicity scores. This structured approach ensures that the model accurately distinguishes between highly immunogenic and non-immunogenic epitopes, facilitating the selection of vaccine targets.

Artificial intelligence (AI) has significantly transformed the landscape of vaccine and immunotherapy development for infectious diseases by enabling data-driven, precise, and scalable approaches to epitope discovery, immune response prediction, and vaccine formulation. Traditional approaches to vaccine development rely on labor-intensive and time-consuming processes involving empirical screening of pathogen proteins, often resulting in limited success and inefficiencies. AI addresses these limitations by incorporating deep learning, machine learning, and natural language processing techniques that integrate diverse immunological and omics datasets to inform rational vaccine design (Anderson et al., 2025). In the context of infectious diseases such as malaria, HIV, tuberculosis, influenza, and dengue, AI models are deployed to predict and prioritize B-and T-cell epitopes with high immunogenic potential, cross-strain conservation, and strong major histocompatibility complex (MHC) binding affinities (Peters et al., 2020). These predictions are based on genomic, transcriptomic, proteomic, and structural data that reflect the evolution of pathogens and the dynamics of host immune responses. Transformer-based deep learning architectures and convolutional neural networks (CNNs) have been used to identify and rank epitopes that are most likely to elicit durable and protective immune responses, as illustrated in Figures 6A,B.

Moreover, AI facilitates reverse vaccinology, an approach that begins with pathogen genome sequences to identify antigens suitable for vaccine development computationally. By leveraging reverse vaccinology pipelines powered by AI, researchers have designed multi-epitope vaccine candidates for complex pathogens, including Plasmodium falciparum (malaria), *Mycobacterium tuberculosis*, and HIV-1 (Hashempour et al., 2024). Recent research has also demonstrated that AI-driven approaches can successfully identify immune response signatures associated with novel vaccine formulations. For example, Chaudhury et al. used machine learning to analyze transcriptomic and proteomic data from vaccine-treated samples, enabling the discovery of biomarkers that predict adjuvant potency and immune pathway activation. This study underscores the power of AI to integrate high-dimensional immune datasets, classify vaccine efficacy outcomes, and inform the rational design of next-generation vaccines with tailored immunostimulatory properties (Chaudhury et al., 2018). A notable application of AI in the context of infectious disease vaccines is the use of computational modeling to predict immune responses to booster immunizations. For example, Shinde et al. conducted an international challenge that benchmarked 49 machine-learning models for predicting individual responses to *Bordetella pertussis* booster vaccines using multi-omics datasets. The study demonstrated that models designed explicitly for the pertussis vaccine task, particularly those incorporating multi-omics integration, dimensionality reduction, and nonlinear modeling, performed significantly better than generic models borrowed from other settings. This underscores the value of AI-guided, context-specific model development in predicting vaccine outcomes and optimizing booster design for infectious diseases (Tascedda et al., 2024). AI also enables the prediction of population coverage by accounting for global HLA polymorphism, thereby ensuring that selected epitopes offer broad protection across ethnically diverse groups. AI also plays a crucial role in modeling the impact of co-infections and immune modulation. In populations affected by latent or concurrent infections, such as HSV-HIV or malaria-HIV, immune responses to one pathogen can dampen or enhance the response to another. AI models simulate these interactions and reveal mechanisms of immune evasion, dysregulation, and synergistic immunopathology that conventional models often miss (Sorci et al., 2013). These insights facilitate the design of combinatorial immunotherapies and vaccines that account for real-world complexity.

Generative models, particularly generative adversarial networks (GANs), have demonstrated effectiveness in refining the design of multi-epitope vaccines. As illustrated in Figure 1D, GANs generate realistic peptide sequences that meet criteria for antigenicity, immunogenicity, MHC binding, and minimal self-reactivity. The resulting constructs are tailored to induce robust CD8^+^ and CD4 + T-cell responses, which are crucial for long-term immunity and pathogen clearance (Li et al., 2021). Importantly, AI is now being used not only for vaccine discovery but also for adaptive optimization during outbreaks. Real-time surveillance data, pathogen mutations, and immune response metrics are fed into AI systems that continuously update antigen selection and vaccine design. This approach has been particularly effective in dealing with rapidly mutating viruses, such as influenza and SARS-CoV-2, and has implications for emerging diseases like the Nipah virus and Zika. Collectively, these advancements underscore AI’s pivotal role in facilitating faster, more targeted, and cost-effective vaccine development pipelines for infectious diseases. As AI algorithms continue to evolve and integrate with high-resolution immunological datasets, their utility in both prophylactic and therapeutic vaccine strategies is expected to expand dramatically.

##### 1.2.1.2 Use of artificial intelligence in pan-coronavirus vaccine development

The COVID-19 pandemic accelerated the application of artificial intelligence (AI) in vaccine research and development on a global scale. Within weeks of the release of the SARS-CoV-2 genome sequence, AI tools were deployed to analyze viral protein structures, identify B-and T-cell epitopes, and model immune responses for candidate vaccine designs (Arora et al., 2021). This rapid integration of AI in vaccine research helped compress the typical development timeline from years to months, showcasing the potential of AI to address urgent global health crises. In recent years, severe outbreaks of SARS-CoV-2 (COVID-19), Ebola, Lassa, Zika, and other emerging viruses have highlighted both the world’s vulnerability to novel pathogens and the urgent need for rapid vaccine innovation frameworks (Olawade et al., 2024; Arora et al., 2021).

AI-powered systems have also been applied to predict cross-reactive memory B-and T-cell responses, which play a critical role in SARS-CoV-2 immunity. Studies show that some individuals exposed to SARS-CoV-2 remain seronegative because of pre-existing cross-reactive CD4^+^ and CD8^+^ T cells, which target conserved non-structural proteins (NSPs) such as those in the replication-transcription complex (RTC), expressed early in the viral lifecycle (Diniz et al., 2022). Additionally, cross-reactive memory B cells have been shown to recognize conserved regions, such as the S2 domain of the spike protein, the nucleocapsid (N) protein, and the membrane (M) protein, facilitating rapid neutralizing antibody responses upon viral exposure (Dobano et al., 2021). Recent AI frameworks have supported these findings by integrating deep learning-based epitope prediction, classification, optimization, and vaccine formulation to identify conserved viral regions that are broadly recognized by human immune memory. This approach allows us to systematically select epitopes that ensure long-term immune memory and broad protection against existing and future variants.

In parallel, AI contributed to real-time genomic surveillance by continuously scanning viral mutation patterns in global sequence databases such as GISAID and modeling their implications for vaccine escape. Reinforcement learning and adaptive modeling helped inform the optimal timing of booster administration, ideal dosing intervals, and heterologous prime-boost strategies, particularly for high-risk populations and immunocompromised individuals (Arora et al., 2021; Lytras et al., 2025). Beyond immunological modeling, AI improved vaccine rollout logistics by optimizing cold-chain infrastructure, anticipating regional demand based on demographic data, and simulating vaccine distribution under multiple disruption scenarios. These insights supported more equitable vaccine access and highlighted the potential of AI to guide end-to-end pandemic response strategies (Mellado et al., 2021).

Taken together, AI-driven approaches to SARS-CoV-2 vaccine development and pandemic mitigation represent a paradigm shift in how vaccines are designed, tested, and deployed. The integration of predictive immunology, population-specific modeling, and real-time response systems now serves as a blueprint for responding to emerging global health threats more effectively and equitably (Arora et al., 2021).

In addition to traditional deep-learning approaches, recent developments have introduced transformer-based architectures and advanced machine-learning tools that significantly enhance epitope prediction and vaccine candidate selection. For instance, EpiBERTope leverages a BERT-based pre-trained language model to predict both linear and structural B-cell epitopes, effectively capturing long-distance protein interactions and thereby improving the interpretability and accuracy of predictions (Park et al., 2023). Meanwhile, Vaxign-ML integrates multiple machine learning algorithms, including deep neural networks, to rapidly evaluate and prioritize vaccine candidates based on antigenicity and host-pathogen interaction features. It has been successfully applied to emerging pathogens such as Nipah and Ebola viruses (Ong et al., 2020a). These tools support the development of pan-variant vaccine strategies by identifying conserved immune targets that remain effective against highly mutable viral lineages (Escalera et al., 2024). To further clarify the breadth and capabilities of AI approaches discussed above, Table 1 summarizes the main classes of AI techniques, their specific applications in vaccine development, key advantages and limitations, and representative studies. This overview provides a structured reference for researchers and practitioners seeking to adopt or compare AI-driven strategies in immunological modeling and vaccine research.

**Table 1:** Summary of key AI approaches in vaccine development.

AI approach	Application	Advantages	Limitations	Representative studies
EpiBERTope (transformer-based)	Predicts linear and structural B-cell epitopes using transformer models	Captures long-distance protein interactions; high interpretability	Requires large training data; computationally intensive	Park et al. (2023)
Ensemble ML (e.g., vaxign-ML)	Identifies and prioritizes vaccine candidates based on antigenicity and host-pathogen features	Integrates multiple algorithms; robust for novel pathogens	May suffer from overfitting in small datasets	Ong et al. (2020a)
NetMHCpan (MHC binding predictor)	Predicts peptide binding affinity to MHC class I and II molecules	High binding prediction accuracy; widely validated	Dependent on HLA allele diversity in training data	Birkir et al. (2020)
VaxiJen	Predicts protective antigens without sequence alignment	Fast and alignment-free; simple implementation	Lower accuracy for complex antigens	Doytchinova and Flower (2007)
IntegralVac (machine learning-based)	Designs comprehensive multivalent epitope vaccines using ensemble learning and multi-feature fusion	Integrates multiple features (antigenicity, immunogenicity, allergenicity); identifies CD4+, CD8 + T-cell, and B-cell epitopes; uses voting-based ensemble methods	May not generalize to all pathogens without sufficient epitope data; performance depends on dataset diversity	Suri and Dakshanamurthy (2022)

##### 1.2.1.3 Use of artificial intelligence in the development of vaccines and immunotherapeutics for cancers

Cancer immunotherapy represents a rapidly evolving frontier in precision medicine; however, it is still hindered by the biological complexity of tumors, their heterogeneity, and their capacity for immune evasion. Artificial intelligence (AI) has emerged as a transformative tool in this domain, enabling researchers to decipher complex tumor-immune dynamics, discover new immunotherapeutic targets, and develop personalized cancer vaccines. AI systems leverage vast datasets, including single-cell RNA sequencing, multi-omics profiles, and digital pathology images, to uncover hidden patterns and generate predictive models that guide therapeutic design and response prediction (Li et al., 2023). One of the most impactful applications of AI in cancer immunotherapy is the identification of tumor-specific neo-antigens. These are peptides that arise from tumor-specific mutations, which are absent in normal tissues. Using deep learning models trained on patient tumor sequences, AI can predict which neoantigens will be strongly presented on major histocompatibility complex (MHC) molecules and elicit robust CD8^+^ T cell responses. This approach has allowed the design of individualized cancer vaccines tailored to each patient’s tumor mutational landscape (Bhinder et al., 2021). Additionally, AI supports the development of shared antigen vaccines by identifying conserved epitopes across tumor types with high immunogenicity and low off-target toxicity. AI also enhances immunotherapy by improving the selection and application of immune checkpoint inhibitors, such as anti-PD-1 and anti-CTLA-4 therapies. As shown in Figure 1A, machine learning models can analyze transcriptomic and spatial tumor data to identify biomarkers predictive of response or resistance, guiding patient stratification and combination therapy strategies (Topalian et al., 2016). Beyond checkpoint blockade, AI predicts the dynamics of memory T cell generation, exhaustion, and reactivation, as illustrated in Figure 1B, facilitating a more accurate prediction of therapeutic durability. Unlike conventional mathematical modeling, AI approaches can simulate immune responses in high-dimensional spaces, incorporating diverse immune cell types, spatial distribution, cytokine gradients, and tumor antigen evolution. These models move beyond oversimplified predator–prey dynamics and instead embrace the nonlinear and context-dependent nature of immunological interactions. AI tools are now being used to simulate how tumors shape their microenvironment through immunosuppressive signals and how therapy modifies this balance.

In vaccine design, AI-driven algorithms have significantly advanced the selection of cancer-associated epitopes. Machine learning platforms screen thousands of peptide candidates for MHC binding, immunogenicity, and mutation frequency. GANs and transformer-based models refine peptide sequences for maximal immunogenic potential while reducing the risk of autoimmune responses. These models also help ensure coverage across diverse HLA types by incorporating supertype-based epitope selection, especially for HLA-A2, HLA-A3, and HLA-B7, enhancing global applicability (Suri and Dakshanamurthy, 2022). Emerging studies also explore the role of AI in combining cancer vaccines with other immunotherapies, such as oncolytic viruses and CAR-T cells. AI can model synergistic effects, predict resistance mechanisms, and guide adaptive dosing regimens (Li et al., 2023; Guedan et al., 2019).

Furthermore, digital pathology integrated with AI is providing insights into the spatial heterogeneity within tumors, enabling clinicians to visualize immune infiltration zones, predict immune cold and hot phenotypes, and localize optimal biopsy and injection sites (Schapiro et al., 2022; Lu et al., 2021). Overall, AI is revolutionizing cancer immunotherapy by enabling highly personalized, adaptive, and efficient therapeutic strategies. As AI systems continue to integrate biological, clinical, and imaging data, their predictive power will enhance not only vaccine efficacy but also overall treatment precision, ultimately improving patient survival and quality of life (Zhang et al., 2023a).

### 1.3 AI-powered epitope prediction: model 1 performance and results

The Antigen/Epitope Prediction Model Architecture is a transformer-based deep learning model designed to predict epitope binding affinity, immunogenicity, and conservation across viral strains. The architecture consists of a preprocessing module that converts viral protein sequences into numerical embeddings using amino acid encoding. This is followed by a transformer-based feature extractor, which captures contextual dependencies among amino acids, improving antigenicity prediction. The multi-task classification module is responsible for predicting binding affinity, conservation, and immunogenicity scores, while an optimization layer enhances predictive confidence by reducing uncertainty. To mathematically characterize the temporal evolution of T-cell proliferation and viral load dynamics in response to infection, we employ a system of nonlinear differential equations (Equations 1–5)

(1) $$f(x)=T(W_{\mathrm{emb}}x+b)$$

Where $W_{\mathrm{emb}}$ is the embedding matrix, which converts input amino acid sequences into numerical representations, x represents the input sequence, consisting of epitope fragments, *T* represents the transformer function, *b i*s the bias term, which ensures better generalization by adjusting predictions independently of input values, especially across diverse protein sequences.

To optimize epitope prediction, the model minimizes a multi-task weighted cross-entropy loss, which combines three key prediction tasks:

(2) $$L=\alpha L_{\mathrm{aff}}+\beta L_{\mathrm{imm}}+\gamma L_{\text{cons}}$$

Where $L_{\mathrm{aff}}$ corresponds to the binding affinity loss function, $L_{\mathrm{imm}}$ represents the immunogenicity classification loss, $L_{\text{cons}}$ measures epitope conservation loss, and *α*, *β*, *γ* are weight coefficients that control the importance of each task.

For binary classification of immunogenic epitopes, we use the binary cross-entropy loss function, ensuring robust differentiation between immunogenic and non-immunogenic peptides:

(3) $$L_{\mathrm{imm}}=-\frac{1}{N}\sum_{i=1}^{N}(y_{i}\log({\hat{y}}_{i})+(1-y_{i})+\log(1-{\hat{y}}_{i}))$$

Where $y_{i}$ is the true immunogenic label (1 for immunogenic, 0 for non-immunogenic), ${\hat{y}}_{i}$ is the predicted probability assigned by the model. *N* is the number of training samples. The model is optimized using Adam (Adaptive Moment Estimation), which dynamically adjusts learning rates, improving convergence speed and preventing unstable updates:

(4) $$m_{t}=\beta_{1}m_{t-1}+(1-\beta_{1})g_{t}$$

(5) $$v_{t}=\beta_{2}v_{t-1}+(1-\beta_{2})g_{t}^{2}$$

Where $g_{t}$ is the gradient at time step *t*, $\beta_{1}\;\text{and}\;\beta_{2}$ are exponential decay rates controlling momentum updates.

Binary cross-entropy loss is particularly suited for this classification task because it penalizes incorrect predictions more heavily and works effectively in imbalanced datasets, which are standard in immunogenicity labeling. This loss function ensures that the model focuses on rare but biologically relevant immunogenic epitopes, enhancing prediction accuracy and reducing false negatives.

To prevent overfitting, the model incorporates dropout layers, randomly deactivating neurons during training. Additionally, early stopping is used to halt training once the validation loss stabilizes, ensuring efficient learning while avoiding excessive computations. To ensure biological relevance and computational efficiency, the model follows a structured pipeline (Figure 6). Initially, input data (protein sequences and epitope labels) are split into training (80%) and testing (20%) sets. Sequences are preprocessed into deep-learning-compatible features. During training and fine-tuning, the model is iteratively optimized and validated. Fine-tuning ensures generalization to unseen data. The model’s performance is assessed using accuracy, precision, recall, and F1-score metrics that are essential for both AI researchers and immunologists. This structured approach maximizes prediction accuracy while preserving interpretability for clinical application.

Recent studies have demonstrated that deep learning methods significantly outperform traditional models in predicting immunogenic epitopes and neoantigens. For instance, Li et al. developed DeepImmuno, a deep learning model that achieved an accuracy of over 0.88 in predicting CD8^+^ T-cell immunogenicity using a large peptide dataset, outperforming traditional machine learning models (Li et al., 2021). Bi et al. proposed an attention-based BiLSTM model for TCR-epitope binding prediction, achieving an C of 0.974 for naïve TCR-epitope and 0.887 for specific binding, outperforming previous models like TCRGP and NetTCR (Bi et al., 2022). Similarly, Jiang et al. developed NeoaPred, a deep-learning framework that incorporates structural and surface features of pHLA complexes, achieving an AUROC of 0.81 and AUPRC of 0.54, which demonstrates strong performance in neoantigen prediction (Jiang et al., 2024). Rajeshwar et al. (2024) presented TCR-H, a support vector machine model that leverages physicochemical features and SHAP-based explainability, achieving AUROCs of 0.87, 0.92, and 0.89 in epitope-hard, TCR-hard, and strict-split settings, respectively, showcasing robust generalizability to unseen test cases (Rajeshwar et al., 2024). Zhang et al. proposed iTCep, a deep learning framework that uses fusion features from sequence encoding strategies to predict peptide–TCR interactions, achieving an AUC of 0.955 on the main test set and over 0.86 on independent datasets (Zhang et al., 2023b). Additionally, Montemurro et al. introduced NetTCR-2.0, a CNN-based model that utilizes paired TCRα and TCRβ chain sequences for predicting TCR–peptide binding, achieving an AUROC of 0.91 and outperforming its predecessor, NetTCR-1.0, as well as other baselines (Montemurro et al., 2021). These findings collectively demonstrate that deep learning models, particularly those leveraging multimodal and paired-chain inputs, are pushing the boundaries of predictive performance in immunogenicity modeling.

#### 1.3.1 Prediction performance and results

**Figure 7:**
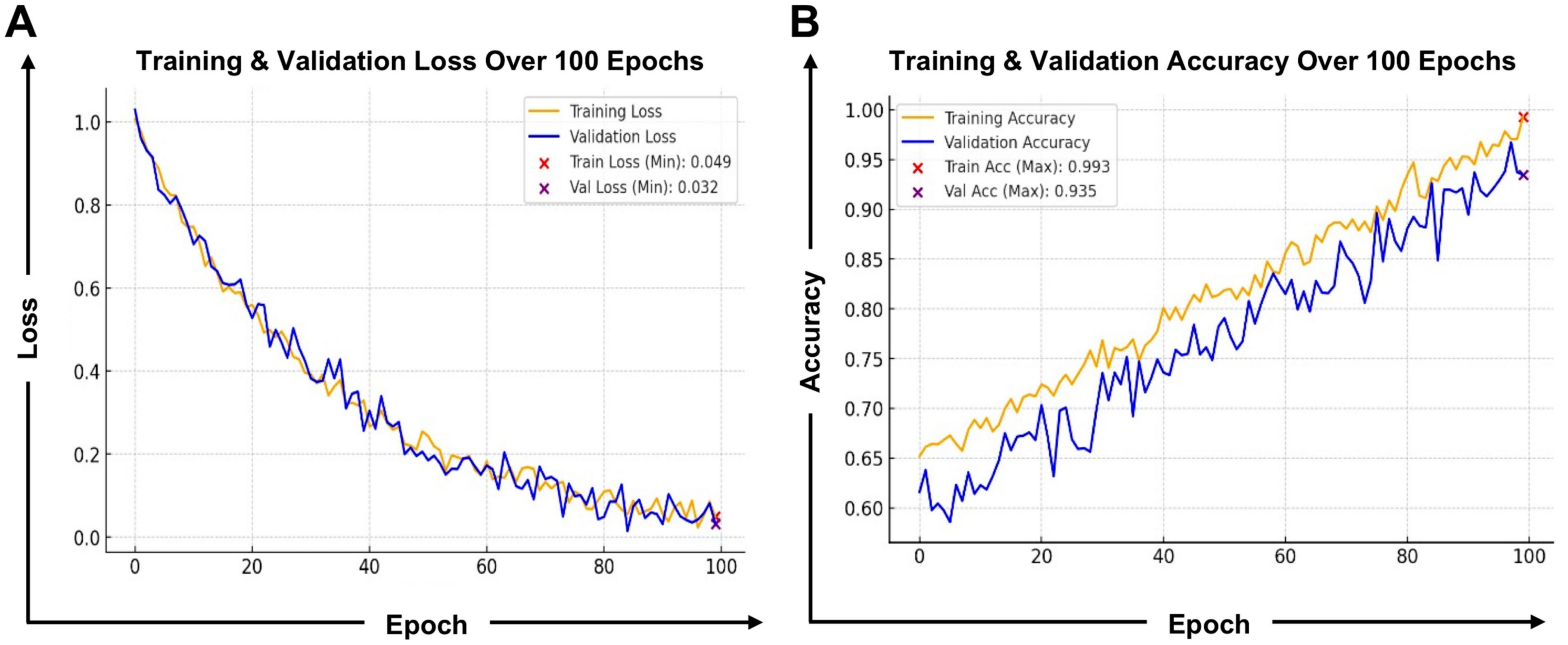
Training and validation performance of the antigen/epitope prediction model: **(A)** Loss and **(B)** Accuracy. This figure presents the training performance metrics of the antigen/epitope prediction model. The left graph displays the training and validation loss over 100 epochs, where both losses decrease progressively, indicating stable optimization. The validation loss stabilizes at 0.032, demonstrating strong generalization without overfitting. The right graph showcases training and validation accuracy, which steadily improved, reaching 0.993 training accuracy and 0.935 validation accuracy. These results confirm that the model effectively learns to recognize key features of immunogenic and non-immunogenic epitopes while maintaining high reliability across validation data. The minimal gap between training and validation curves highlights robust model performance, ensuring applicability for real-world antigen screening.

The Antigen/Epitope Prediction Model (Figure 7A) employs a transformer-based deep learning approach to predict epitope binding affinity, immunogenicity, and conservation across various viral strains. The model was trained over 100 epochs (Figure 7), achieving a training accuracy of 0.993 and a validation accuracy of 0.935. Training loss stabilized at 0.049, while validation loss remained at 0.032, indicating strong generalization capability. These metrics demonstrate the model’s high efficiency in learning patterns within antigen sequences, enabling it to distinguish immunogenic from non-immunogenic regions with high confidence. Model performance (Figure 7) shows impressive learning behavior. The classification report (Supplementary Figure S1B) indicates a precision of 0.97, a recall of 0.98, and an F1-score of 0.98. This means that when the model predicts an epitope as immunogenic, it is correct 97% of the time, minimizing false positives. Its high recall ensures nearly all true epitopes are identified, avoiding the loss of promising candidates.

The confusion matrix (Supplementary Figure S1A) supports these results, showing 2,448 true negatives and 2,434 true positives, with only 65 false negatives and 53 false positives. This high specificity and sensitivity are vital in vaccine research, where overlooking an immunogenic epitope (false negative) or selecting a weak candidate (false positive) can hinder vaccine development. The model also identifies top-ranking CD8^+^ T-cell epitopes based on binding affinity and conservation scores (Supplementary Figure S2B). The epitope YLQPRTFLL (HLA-A*02:01) showed the most substantial potential, with a binding affinity of 35.7 nM and a conservation of 94.5%. Similarly, TTDPSFLGRY (HLA-B*07:02) achieved high affinity at 22.1 nM, making it broadly relevant across populations. A strong binding affinity ensures that antigen-presenting cells (APCs) effectively present antigens to T-cells, enabling a robust immune response. By identifying epitopes with both high affinity and conservation, the model streamlines experimental validation.

Reliable evaluation of AI models in immunology requires rigorous and standardized validation frameworks. Commonly used metrics include accuracy, precision, recall, and the F1 score (Supplementary Figure S2B), as well as the area under the receiver operating characteristic curve (AUC-ROC) (Figure 7) for classification tasks such as epitope binding prediction. For regression-based predictions (e.g., peptide–MHC affinity scores), mean squared error (MSE) and R^2^ values are typically used. Calibration curves help assess the reliability of predicted probabilities, while SHAP values, attention maps, and other explainability tools are increasingly used to interpret model outputs. Benchmark datasets, such as the IEDB database, NetMHCpan ligand datasets, and epitope prediction challenges, serve as gold standards for comparing model performance across studies. Transparent reporting of these metrics is essential for reproducibility and regulatory trust.

#### 1.3.2 Epitope immunogenicity ranking and analysis

The epitope immunogenicity ranking (Supplementary Figures S2A,B) highlights the model’s capacity to prioritize antigenic regions likely to trigger immune responses. YLQPRTFLL scored highest (~0.98), suggesting strong potential for CD8^+^ T-cell activation. TTDPSFLGRY and NQKLIANQF followed, each with scores greater than 0.90, making them excellent candidates for broad HLA population coverage. Conversely, SPRWYFYYL and LSPRWYFYY had lower scores (~0.88–0.89), suggesting a reduced capacity to initiate a strong immune response. These patterns align with known immunogenicity data and validate the model’s predictive ability. By focusing on epitopes with high immunogenicity, the model enhances vaccine target selection, thereby minimizing the allocation of resources to weak candidates. The observed correlation between predicted binding affinity and immunogenicity scores confirms the model’s strength in selecting potent immune triggers. High-ranking epitopes also indicate a strong interaction potential with antigen-presenting cells (APCs), which is crucial for long-term immune memory and vaccine durability. In conclusion, this transformer-based model 1 presents a robust, explainable, and biologically grounded framework for epitope prediction, offering real-world value for vaccine researchers, immunologists, and AI scientists alike.

### 1.4 Induction and maintenance of protective memory CD8+ T cells: what AI modeling assumed vs. what experimental data proved or disproved

Understanding the mechanisms governing CD8^+^ T cell activation, survival, and long-term maintenance has been a significant focus in immunology for years (Wherry and Kurachi, 2015). Traditional computational models assumed that CD8^+^ T cell expansion required continuous antigenic stimulation. In contrast, recent AI-driven immune simulations have shown that a single antigen encounter can trigger a program of proliferation and differentiation, resulting in the generation of both effector and memory CD8^+^ T cells. AI-based modeling of CD8^+^ T cell kinetics has been instrumental in identifying key activation markers, proliferation rates, and survival factors. Unlike conventional models, AI frameworks dynamically adapt to experimental data, refining predictions on memory CD8^+^ T cell function in response to known epitopes. A comparative analysis of AI-driven and experimental models has revealed that previous mathematical models have failed to accurately predict HSV-specific CD8^+^ T cell responses in mice, rabbits, and humans (Girel et al., 2019). CD4^+^ T helper cells play a crucial role in priming CD8^+^ T cells, facilitating both primary immune responses and the development of protective memory CD8^+^ T cells. AI-driven models highlight that CD4^+^ T cell interactions during priming encoding memory potential, enabling autonomous secondary expansion upon antigen re-encounter. Experimental data have confirmed that CD8^+^ T cells primed in the absence of CD4^+^ T cells fail to undergo secondary expansion, although they retain cytotoxic activity. AI-based predictive analytics have been utilized to model CD4^+^ T cell help requirements in various infection scenarios, thereby refining our understanding of immune memory formation.

Previous mathematical models argued that CD8^+^ T cells could clear infections without CD4^+^ T cell help, provided that the viral replication rate remained low. However, AI-driven simulations incorporating longitudinal immune response data have shown that CD4^+^ T cell help is essential for sustained viral clearance. AI-enhanced models suggest that in the absence of help, CD8^+^ T cells reduce viral loads temporarily but fail to prevent resurgence. This is due to insufficient memory T cell reactivation, which is critically dependent on antigen presentation and cytokine signaling mediated by CD4^+^ T cells. In addition to CD4^+^ T cells, dendritic cells (DCs) have been identified as key players in memory CD8^+^ T cell priming and maintenance (Figure 5C). AI-driven models have also refined our understanding of the differences between central and effector CD8^+^ T cells, showing that their fate is pre-programmed by early priming signals. These insights have significant implications for the development of CD8^+^ T cell-based vaccines, providing guidance on optimal strategies for antigen exposure and presentation. AI simulations have confirmed that depleting CD4^+^ T cells at the priming stage results in impaired CD8^+^ T cell memory formation, but interestingly, late-stage depletion has minimal effects (Laidlaw et al., 2014). Other studies contradict this, showing that CD4 + T cell help can occur later in the development of the immune response (Shedlock and Shen, 2003). AI frameworks have reconciled these discrepancies by modeling heterogeneous immune environments, demonstrating that CD4^+^ T cell support can be context-dependent.

Recent AI-driven studies have revealed that CD4 + T cell help is critical in preventing CD8^+^ T cell apoptosis, particularly via the regulation of tumor necrosis factor-related apoptosis-inducing ligand (TRAIL). AI-powered immune simulations suggest that CD4^+^ T cells regulate IFN-*γ* secretion and local chemokine expression, which are essential for CD8^+^ T cell migration to infected tissues. This directly contradicts earlier mathematical models that suggested CD8^+^ T cell expansion was independent of CD4^+^ T cell-mediated migration signals. AI models now incorporate spatial variables, accurately predicting cellular migration dynamics and antigenic stimulation requirements. AI-driven research has also challenged previous programmed division models of CD8^+^ T cell expansion. Traditional models assumed that CD8^+^ T cell division was independent of antigenic stimulation, continuing even at low viral loads. AI simulations incorporating real-world patient data suggest a different scenario: while early CD8^+^ T cell divisions are antigen-independent, continued expansion and viral clearance require persistent antigen exposure and co-stimulatory signaling. AI models predict that programmed divisions are optimized to balance viral clearance and immune homeostasis, ensuring effective pathogen elimination while preventing excessive immunopathology. Unlike static mathematical models, AI-based frameworks are capable of real-time adaptive learning, adjusting predictions in response to emerging experimental data. AI simulations have provided more accurate insights into viral clearance mechanisms, leading to refined vaccine designs. AI-driven approaches now integrate multi-omics datasets, single-cell RNA sequencing, and immunophenotyping data, ensuring that computational models align with experimental observations.

AI models used in immunology and vaccine research are prone to overfitting, particularly when trained on small, biased, or non-representative datasets. Overreliance on computational predictions without thorough validation may lead to clinically unsafe or misleading results. Therefore, these models must be interpreted within the context of immunological knowledge and clinical practice. Incorporating expert review during model development and post-analysis, along with rigorous testing on diverse datasets, is crucial to ensure the safety, accuracy, and translational value of the model.

Biomedical datasets used in AI-driven immunology and vaccine research often suffer from various forms of bias. These include the underrepresentation of specific demographic groups, limitations in geographic sampling, and annotation inconsistencies resulting from human error or inadequate clinical guidelines. Such biases can impair model generalizability and risk reinforcing health disparities. Addressing this challenge requires careful dataset curation, transparent documentation of data provenance, and, where possible, the use of federated learning and diverse multi-center datasets to minimize overfitting to a specific sub-population.

The integration of AI into CD8^+^ T cell research has significant implications for the design of next-generation vaccines and immunotherapies. AI-driven models have already identified optimal antigen exposure strategies, cytokine modulation approaches, and co-stimulatory molecule enhancements to maximize long-term immune protection. Future research will focus on further refining AI algorithms to predict patient-specific immune responses, paving the way for precision immunotherapy and personalized vaccine design.

### 1.5 The use and abuse of AI-driven modeling in cancer vaccines and immunotherapies

Artificial intelligence (AI) has made significant advancements in understanding cancer immunity mechanisms and optimizing vaccine design strategies. AI-driven predictive models, adapted from previous frameworks designed for viral infections, are now being utilized to analyze cancer-immune interactions (Huang et al., 2020). These AI models account for tumor progression, immune suppression dynamics, and adaptive immune responses, refining predictions on how the immune system combats tumors. Unlike traditional static models, AI frameworks continuously learn from real-time immunological data, making them superior in predicting tumor-immune system interactions. AI-driven models simulate the interplay between tumor growth and immune response, capturing how tumors evade immune detection while simultaneously activating CD8^+^ T cells and other immune components (Cess and Finley, 2020). These models integrate multi-modal datasets that include genomic, proteomic, and immunological parameters, ensuring that predictions align with real-world immune responses. Unlike early computational models, which oversimplified immune responses, AI-driven frameworks incorporate key players such as regulatory T cells (CD4^+^CD25^+^), antigen-presenting cells (APCs), and cytokine networks, offering a comprehensive perspective on immune dynamics in cancer.

Traditional models have assumed that cancer cells stimulate immune proliferation while simultaneously impairing immune responses, leading to highly dependent outcomes on specific equations. AI models refine this understanding by continuously training on experimental data, highlighting novel immune escape mechanisms, T cell exhaustion pathways, and tumor antigen presentation strategies. These models have revealed how tumor-specific immune suppression affects vaccine efficacy and the success of immunotherapy, leading to more precise treatment strategies. AI-driven cancer immunology modeling predicts several possible immune response outcomes: (i) an effective CD8^+^ T cell response that establishes equilibrium, keeping tumor growth in check; (ii) immune system failure, allowing tumor progression due to excessive immune suppression and tumor cell resistance; or (iii) a dynamic state where the immune system and tumor continuously adapt, requiring sustained therapeutic intervention. Unlike conventional models, AI-driven approaches quantify the quality of immune responses, not just the number of T cells, ensuring greater accuracy in predicting tumor clearance potential.

One of the primary weaknesses of traditional models was their reliance on T cell population numbers alone, without considering the functionality and migration patterns of immune cells. AI-driven models correct this by incorporating spatial and temporal immune dynamics, showing that T-cell homing to tumor sites is just as critical as their activation levels. AI-driven simulations suggest that dendritic cells and regulatory T cells play crucial roles in determining long-term immune memory and the sustainability of adaptive responses (Figure 5B). AI has also enhanced our understanding of tumor resistance mechanisms. Unlike earlier models that treated tumor cells as a uniform population, AI-driven frameworks integrate heterogeneous tumor subpopulations, including drug-resistant and immune-sensitive variants. These models accurately predict tumor cell evolution during treatment, enabling the optimization of combination immunotherapies, immune checkpoint blockade strategies, and personalized T-cell therapies. Early computational models attempted to link immune response strength to tumor burden, often using oversimplified growth-decline equations that failed to capture the real-world dynamics of treatment accurately. AI-driven models overcome this limitation by incorporating single-cell sequencing data, immune evasion modeling, and simulations of treatment responses. AI-driven predictions suggest that early immune activation promotes long-term tumor suppression, whereas delayed or weak responses are associated with a poor prognosis and increased resistance to therapy. Figure 5A illustrates how Artificial Intelligence (AI) plays a crucial role in understanding and optimizing tumor-immune interactions, particularly in the regulation of checkpoint inhibitors, which have transformed cancer immunotherapy. Tumor cells often evade immune detection by suppressing T cell activation, a process mediated through immune checkpoints such as PD-1/PD-L1 and CTLA-4 pathways (Yoo et al., 2025). Checkpoint inhibitors, such as anti-PD-1 and anti-CTLA-4 monoclonal antibodies, help restore T-cell activity against tumor cells. However, determining the most effective checkpoint blockade strategies requires advanced computational approaches, and this is where AI-driven models have shown significant promise. Figure 5A illustrates this mechanism by showing how tumor cells inhibit T cells (immune suppression) while AI-driven models predict and optimize checkpoint blockade strategies. AI aids in identifying patient-specific responses to checkpoint inhibitors, enhances biomarker discovery, and improves the efficacy of combination immunotherapies (Yoo et al., 2025). Moreover, AI-assisted analysis of multi-omics data (genomics, transcriptomics, proteomics) enables personalized immunotherapy approaches, ensuring better treatment outcomes with reduced toxicity (Li et al., 2024).

The role of Artificial Intelligence (AI) in understanding and optimizing memory T cell differentiation, as illustrated in Figure 5B, presents a key process in adaptive immunity. Naïve T cells, upon encountering an antigen, undergo activation and differentiation into effector T cells, which mediate immediate immune responses. A subset of these effector T cells subsequently transitions into memory T cells, which provide long-term immune protection and faster responses upon reinfection. AI-driven approaches enhance this differentiation process by analyzing large-scale immunological datasets, predicting T-cell activation dynamics, and optimizing memory cell formation for vaccine development and immunotherapy applications (Li et al., 2024). In the figure, AI contributes to three key stages of T-cell differentiation. First, it predicts activation by analyzing antigen exposure and T cell receptor (TCR) signaling, allowing for a deeper understanding of when and how naïve T cells transition into effector T cells. Second, AI analyzes differentiation markers, evaluating gene expression and molecular pathways that distinguish short-lived effector T cells from long-lasting memory T cells, which is critical in immunotherapy and vaccine design. Finally, AI plays a crucial role in optimizing memory formation by refining models that predict T cell persistence and longevity, ensuring that immune memory is robust and effective for long-term protection. This is particularly valuable in the development of next-generation vaccines and personalized cancer immunotherapies (Garg et al., 2024). AI-based models, trained on single-cell RNA sequencing (scRNA-seq) and epigenetic data, enable researchers to identify key molecular pathways that regulate immune memory. Recent work by van Dorp introduces a variational deep-learning framework that jointly models the phenotypic heterogeneity and temporal dynamics of lung-resident memory CD4^+^ and CD8^+^ T cells (van Dorp et al., 2025). Their approach integrates stochastic variational inference with flow cytometry data, enabling the discovery of novel insights into the persistence and differentiation of memory T cells over time. These AI-driven insights offer new avenues for designing durable vaccine strategies, refining T-cell-based immunotherapies, and deepening our understanding of chronic infections and immune exhaustion. By integrating AI into immunology, researchers can develop more precise, data-driven treatment strategies that enhance immune responses and improve long-term health outcomes.

Unlike traditional models that assume a linear relationship between tumor growth and immune response, AI simulations have revealed that immune-tumor interactions are inherently non-linear, influenced by T-cell infiltration rates, antigen exposure, and regulatory immune pathways. Earlier models incorrectly suggested that immune failure was solely due to antigen depletion. In contrast, AI-driven insights reveal that immune exhaustion and the expansion of regulatory T cells are the primary factors contributing to immune escape. AI-driven modeling has been crucial in guiding the development of cancer vaccines t and optimizing immunotherapy protocols. By integrating deep learning, multi-omics data, and patient-specific immune profiling, AI-driven approaches can predict personalized success rates for immunotherapy. Unlike previous models that focused only on T cell proliferation rates, AI simulations emphasize the importance of immune memory retention, migration dynamics, and metabolic fitness, leading to more precise and individualized cancer treatment plans. Ultimately, AI-driven modeling bridges the gap between computational immunology and real-world clinical applications, providing unparalleled insights into cancer-immune system interactions and optimizing therapy. Future research will focus on refining AI frameworks to integrate real-time clinical trial data, ensuring that AI-generated predictions translate into clinically actionable strategies. By leveraging AI, researchers can accelerate breakthroughs in cancer immunotherapy, precision medicine, and vaccine development, ultimately improving outcomes for cancer patients worldwide.

### 1.6 Future directions in AI-driven vaccine and immunotherapeutic development

Despite recent breakthroughs, AI-driven vaccine development remains in its early stages and faces several key challenges that define future research priorities. A significant obstacle is the fragmentation and inconsistency of available data, which impacts the accuracy and scalability of AI-driven vaccine models. The development of effective vaccines relies on extensive datasets, including genomic sequences, protein structures, immune response metrics, and clinical trial results. However, these datasets often suffer from incompleteness, bias, and a lack of standardization, which limits the ability of AI models to generate robust and generalizable predictions across diverse populations. Moving forward, future efforts must prioritize harmonizing global data collection methodologies, fostering international data-sharing collaborations, and establishing standardized frameworks tailored for AI-driven immunology research. Another critical future direction involves optimizing the computational infrastructure for AI-based vaccine development. Cutting-edge deep learning algorithms require substantial processing power, memory resources, and access to high-performance computing (HPC) clusters or cloud-based AI infrastructure. However, these resources are not universally accessible, particularly in low-resource research settings. Future strategies must focus on expanding equitable access to AI tools, developing more computationally efficient models, and implementing distributed learning techniques to democratize vaccine innovation globally.

Improving model interpretability will also remain a central priority. Many deep-learning vaccine prediction models operate as black boxes, making it difficult for researchers to fully understand the rationale behind specific predictions. This lack of transparency raises concerns about the model’s reliability, potential biases, and the biological relevance of AI-generated vaccine candidates. To enhance trust in AI-driven vaccine solutions, the scientific community is actively developing explainable AI (XAI) techniques (Garg et al., 2024), including feature attribution methods, visualization tools, and interpretable surrogate models. These approaches aim to increase transparency and align AI-generated predictions with immunological principles and experimental validation.

Furthermore, interdisciplinary collaboration between computational biologists, immunologists, clinicians, and data scientists will be essential for refining AI-driven vaccine strategies. By combining domain expertise with AI advancements, researchers can improve vaccine candidate selection, refine AI-driven immune response models, and address data inconsistencies. Strengthening such partnerships will help ensure that AI-generated insights translate into actionable and biologically meaningful vaccine designs.

Ethical considerations, model fairness, and regulatory readiness will also be critical moving forward. Establishing guidelines for equitable vaccine distribution, mitigating algorithmic biases, and maintaining data privacy will be crucial in building trust and accelerating the integration of AI into real-world applications. Future directions will also involve aligning AI innovation with global health priorities, ethics, and policy standards to enhance vaccine accessibility and improve public health outcomes worldwide.

### 1.7 Ethical considerations in AI-driven vaccine development

The growing integration of AI in vaccine research raises several critical ethical considerations. First, data privacy is a primary concern, especially when models are trained on sensitive clinical or genomic data. Ensuring that patient-level information is anonymized and securely handled is critical. Second, algorithmic bias remains a pressing issue. AI models trained on unbalanced or demographically skewed datasets may produce inequitable outcomes, underrepresenting certain ethnicities or regions in epitope prediction or immune response modeling. Third, equitable access to AI-derived vaccines must be prioritized. If advanced AI tools are only accessible to high-resource settings, global vaccine equity will worsen. Responsible AI implementation should include open-access platforms, transparent model interpretability, and public-private collaboration to ensure ethical oversight. As AI becomes further embedded in biomedical innovation, aligning its deployment with principles of fairness, accountability, and global health equity will be essential.

Despite their promise, AI-driven approaches are not without limitations. Predictive accuracy can vary significantly depending on the quality and representativeness of the training data. Moreover, AI models may produce unreliable outputs when applied to novel or out-of-distribution inputs. In some cases, traditional experimental methods may be more reliable, especially for validating immunogenicity and efficacy. Additionally, time-to-result for AI systems is not always shorter; preprocessing, model tuning and iterative retraining can be time-intensive. Thus, AI methods should be viewed as complementary to, rather than replacements for, traditional approaches.

Similarly, while AI holds the potential to reduce reliance on animal testing in early-stage immunological research, current models remain adjunctive rather than fully autonomous. In silico simulations can support hypothesis generation and candidate screening, but experimental validation, including *in vitro* assays and *in vivo* models, remains essential for confirming immunogenicity, toxicity, and efficacy. Overstating the standalone potential of AI risks premature deployment or overconfidence in unverified models. It is, therefore, critical to position AI as a complementary tool within a broader experimental and regulatory framework.

### 1.8 The future of AI-driven preclinical *in vivo* testing of drugs, vaccines, and immunotherapeutics

**Figure 8:**
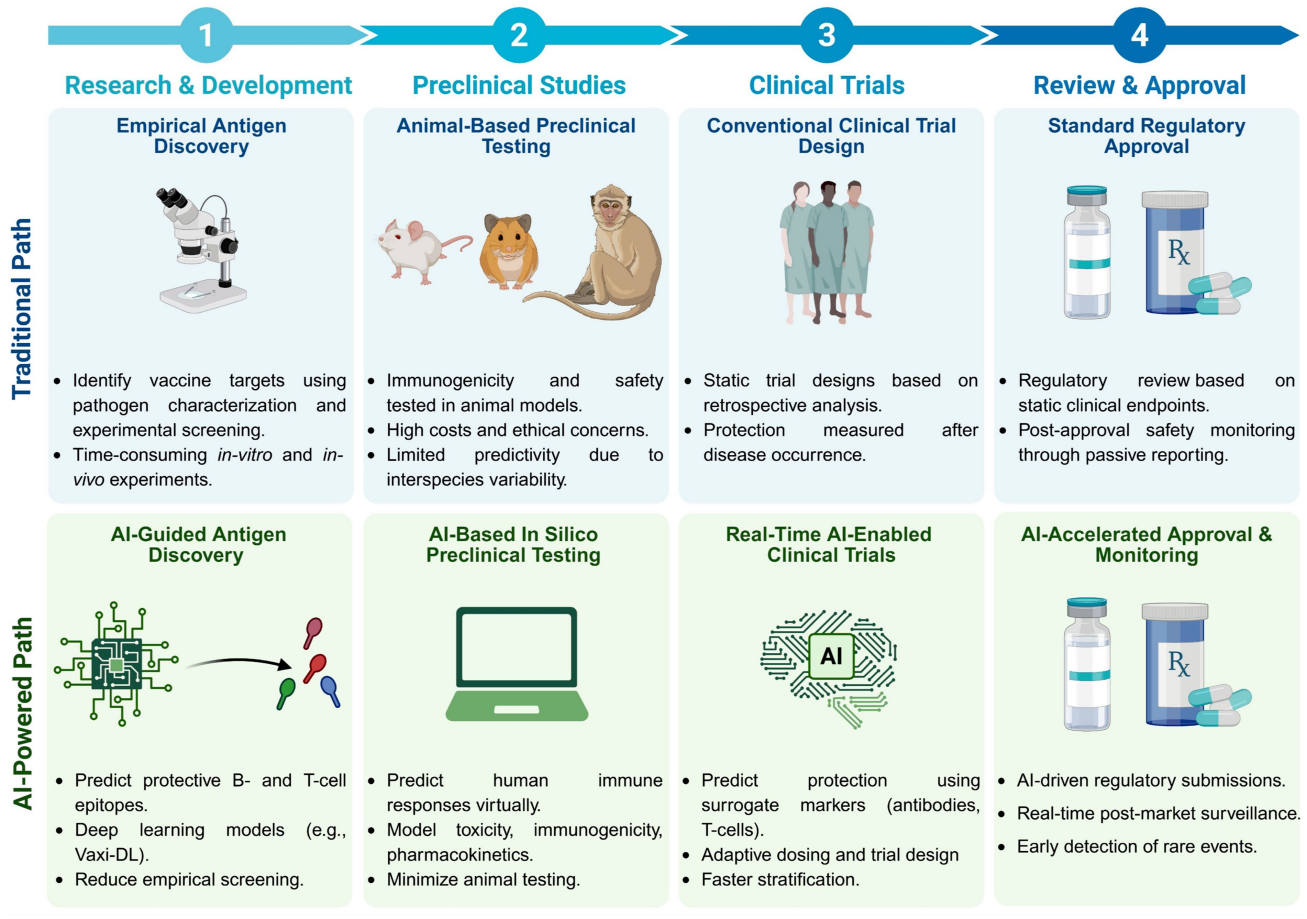
Artificial intelligence is revolutionizing the pre-clinical and clinical development of vaccines and immunotherapeutics. The traditional pathway (top panels) relies heavily on empirical antigen discovery, animal-based preclinical testing, conventional clinical trial designs, and retrospective regulatory evaluation, which can lead to ethical concerns, interspecies variability, and prolonged development timelines. In contrast, the AI-powered pathway (bottom panels) illustrates how emerging computational models can replace or reduce the need for animal preclinical testing, as endorsed by the United States FDA under the Modernization Act 2.0. AI-driven platforms leverage deep learning to predict protective antigens, simulate human immune responses *in silico*, and enable real-time immune-bridging strategies during clinical trials. These advancements accelerate candidate selection, optimize clinical trial design, predict protection based on immune markers, and enhance the speed and precision of regulatory approval and post-market surveillance.

The traditional reliance on animal models for preclinical testing of drugs, vaccines, and immunotherapeutics is being challenged by the emergence of AI-driven computational models. These approaches promise to overcome significant limitations of animal studies, including interspecies differences, ethical concerns, high costs, and long development timelines. As illustrated in Figure 8, artificial intelligence platforms leveraging deep learning, reinforcement learning, and generative models can simulate complex biological systems at the molecular, cellular, and tissue levels, providing predictive insights into drug efficacy, toxicity, and immunogenicity (Zushin et al., 2023; Jimenez-Luna et al., 2021). The recent FDA Modernization Act 2.0 officially recognizes non-animal technologies, including AI models, as acceptable alternatives for specific preclinical evaluations (Zushin et al., 2023). Innovative systems such as Vaxi-DL, a deep learning-based platform for vaccine antigen prediction, demonstrate how in silico models can prioritize vaccine candidates with high sensitivity and accuracy, significantly reducing dependence on animal testing (Rawal et al., 2022). Furthermore, emerging AI frameworks are capable of modeling pharmacokinetics, drug metabolism, and host immune responses, enabling the rapid virtual screening of therapeutic candidates before clinical trials (Guo et al., 2023).

Regulatory agencies such as the U.S. Food and Drug Administration (FDA) are increasingly recognizing the value of AI-driven modeling tools in preclinical evaluation. For instance, the DILIsym platform, a quantitative systems toxicology model, has been used to simulate and predict drug-induced liver injury and has been referenced in multiple New Drug Application (NDA) and Investigational New Drug (IND) submissions accepted by the FDA (Watkins, 2020). Similarly, the FDA’s Office of Clinical Pharmacology has developed Virtual Clinical Trial (VCT) platforms that use population PK/PD models to predict patient-specific drug exposures. These tools have been used internally to guide regulatory decision-making for drug labeling and dose optimization (Samei et al., 2020). Such examples illustrate the growing regulatory acceptance of AI models in formal drug development pipelines.

These AI-enabled platforms integrate diverse data modalities, including omics profiles, organoid models, and electronic health records, to produce human-specific predictions that are often more relevant than results obtained from animal experiments. As a result, AI-driven preclinical models not only offer ethical and financial advantages but also promise to enhance the translational success rate of novel drugs and immunotherapies, accelerating their path to clinical application.

One critical future priority is the ability of AI models to dynamically incorporate emerging biological data, such as pathogen mutations (e.g., SARS-CoV-2 variants), updated immune signatures, or new clinical trial outcomes. Real-time modeling requires adaptive learning frameworks that include automated data pipelines, regular retraining schedules, and mechanisms for integrating experimental feedback. In practice, model retraining can be triggered periodically (e.g., weekly or monthly) or event-driven (e.g., when a significant number of new sequences or immune profiles are available). For example, as new variants emerge, epitope prediction and immunogenicity models must be updated to reflect antigenic drift and shifting population immunity. These updates often rely on feedback loops that incorporate lab assay results, population-level immunogenicity data, and in silico validation to refine model parameters. Establishing robust, automated retraining workflows can enhance responsiveness, accuracy, and public health relevance in dynamic settings involving infectious diseases.

### 1.9 Enabling real-time *in vivo* modeling of immune bridging and prediction of protection in clinical trials

Artificial intelligence (AI) and deep learning (DL) are increasingly transforming clinical trial methodologies by enabling real-time *in vivo* modeling of immune responses, thereby facilitating immune-bridging strategies and predictive protection assessments. Instead of relying solely on traditional endpoints, such as disease occurrence, AI models now enable researchers to simulate human immune dynamics and predict vaccine efficacy based on surrogate markers, including antibody titers, T-cell responses, and cytokine profiles. Recent advances in AI-driven approaches have shown that models trained on multi-omics datasets, immune phenotyping, and clinical biomarkers can identify correlates of protection with high accuracy, dramatically accelerating clinical trial timelines (Mak and Pichika, 2019) (Figure 8). For instance, deep learning models have been successfully employed to analyze longitudinal immunological data and forecast protection levels across diverse demographic groups, improving the stratification and adaptive design of clinical studies (Vamathevan et al., 2019). Moreover, AI-based platforms are being integrated into clinical trial infrastructures to facilitate real-time data monitoring, optimize dosing strategies, and dynamically adjust trial protocols based on predictive safety and efficacy outcomes (Rawal et al., 2022; Guo et al., 2023). Such capabilities are crucial for vaccine development, particularly in rapidly evolving scenarios, such as the emergence of new infectious diseases and viral variants (Mak and Pichika, 2019). By replacing retrospective analysis with real-time immune-bridging predictions, AI holds the potential to enhance the precision, speed, and ethical conduct of clinical trials, leading to faster and more reliable delivery of vaccines and immunotherapeutics (Zushin et al., 2023).

## 2 Conclusion

The relationship between wet-lab immunological research and artificial intelligence (AI) modeling is both complex and critical. Immunologists often view AI researchers as theoreticians who make oversimplified assumptions, abstracting biological processes into computational models that may not fully capture the intricacies. Integration of AI into immunology is paving the way for rapid, precision-driven vaccine development. AI-powered models can predict cross-reactive immune responses, optimize multi-epitope vaccine candidates, and streamline clinical trials. As demonstrated in AI-driven vaccine research, integrating AI with experimental immunology enables real-time adaptation to emerging pathogens, ensuring scalable and effective vaccine solutions. Future research should focus on refining AI explainability, enhancing multimodal data integration, and promoting AI-immunology collaborations to accelerate global vaccine development efforts. Conversely, AI researchers sometimes view immunologists as hard-nosed experimentalists who overlook the complex, non-linear interactions within the immune system. AI-driven models capture these non-linear relationships and generate insights that may not be evident through traditional wet-lab approaches. AI researchers believe that computational models can validate immunological hypotheses and distinguish between competing theories of immune response mechanisms. Given these perspectives, interdisciplinary collaboration between AI researchers and immunologists is crucial for advancing scientific knowledge. AI models should not attempt to simulate entire immune systems in exhaustive detail; instead, they should focus on identifying meaningful biological patterns. Moreover, AI-driven conclusions must be experimentally validated to avoid the pitfalls of algorithmic overfitting and reliance on biased datasets. Misapplications of AI in immunology often stem from using conventional machine learning techniques in dynamic, non-linear biological processes without appropriate adaptation. Immunologists must acquire foundational knowledge of AI to effectively evaluate computational models and select suitable methods for their research.

AI modeling in immunology often employs a reductionist approach, whereby specific biological processes are isolated and formalized into computational algorithms. While this allows for detailed analysis of immune interactions, it can sometimes result in models that fail to capture the complexity of immune dynamics. Despite AI’s ability to analyze immune system behaviors and predict outcomes, translating these insights into real immunological applications remains a challenge.

Although AI-driven modeling has demonstrated impressive capabilities in simulating immune responses, epitope prediction, and vaccine optimization, many of these frameworks are still at the proof-of-concept or simulation level. The real-world application of these models remains limited by the lack of large-scale experimental and clinical validation. Translating AI insights into clinical practice requires rigorous prospective studies, multi-center validations, and continuous feedback from biological assays to ensure reliability and reproducibility. As such, we emphasize that current AI models should be considered complementary to empirical approaches rather than replacements until further validation is achieved.

Some models suffer from excessive parameterization, which can obscure biological relevance and lead to misinterpretations of experimental data. Additionally, the effectiveness of AI models is often limited by the quality and completeness of the input data. Poorly annotated datasets or insufficient immunological information can lead to inaccurate computational predictions. To enhance AI integration in immunology, effective communication between AI researchers and immunologists is essential. AI researchers must engage with experimental immunology literature, understand the limitations of immunological assays, and design models that align with real-world biological mechanisms. AI models often prioritize efficiency by minimizing the number of variables considered, frequently reducing immune system simulations to two-dimensional dynamic models (e.g., protein concentration over time). Few AI models incorporate spatial dimensions of immune responses, as spatial modeling significantly increases computational complexity.

Furthermore, immunological spatial datasets remain limited, making AI-driven spatial modeling more challenging. Immunologists must emphasize that both the position and movement of immune-related molecules are critical for any AI-driven model. While AI-driven immunology often focuses on cellular interactions, future models should integrate the immune tissue environment and the dynamics of both lymphoid and non-lymphoid organs. AI-driven models that consider only immune cell counts may overlook crucial spatial interactions that impact immune responses. The lack of understanding among AI researchers regarding immune cell dynamics, protein transport pathways, and tissue-specific interactions remains a significant challenge.

AI-based models can incorporate multivariate immune cell populations; however, increasing the model’s complexity may compromise its interpretability and usability. AI researchers often favor simpler models that yield interpretable outputs, whereas immunologists require models that accurately reflect the behaviors of the real immune system. This trade-off must be carefully managed to ensure AI models remain useful and clinically relevant. Many AI-driven models make naïve assumptions that oversimplify the complexity of immune responses. For example, some AI models treat T cell immunity as consisting solely of effector CD4^+^ or CD8^+^ T cells, failing to account for regulatory T cells (CD4^+^CD25^+^), which play a crucial role in balancing immune activation and suppression. While similar AI modeling approaches have been successfully applied in fields like chemical engineering (e.g., modeling population dynamics in industrial bioreactors), their application to immunology requires additional complexity. The primary goal of AI modeling in immunology is to generate hypotheses and identify key experimental variables. AI models should guide experimental immunologists toward promising research directions by highlighting immune mechanisms that warrant further investigation. Testing and refining AI-driven models with experimental data will improve their reliability and applicability in immunology research. AI has been increasingly applied in studying the interactions between HIV, HSV, and immune target cells. While AI-driven models have provided valuable insights into AIDS and herpes disease progression, most models assume that target cells are infected with a single virus (e.g., either HIV or HSV). This assumption fails to reflect real-world co-infection scenarios, where multiple viruses can simultaneously infect cells. AI-driven models must evolve to simulate better multi-pathogen interactions and the immune system’s response to complex infections. To account for co-infections, AI modeling must integrate data on how different pathogens interact within the immune environment. Traditional AI models struggle to capture the full dynamics of viral replication, immune evasion strategies, and immunotherapeutic interventions. Future models must incorporate real-world biological complexities to simulate disease progression and treatment outcomes accurately.

AI researchers serve as translators of experimental immunology findings, converting empirical data into computational frameworks. However, for AI models to be meaningful, AI researchers must develop a solid foundation in modern immunological principles. This will enable them to formulate biologically relevant hypotheses and create models that accurately reflect the behavior of the actual immune system. Within AI research, there are distinct groups: (a) theoretical AI researchers who focus on algorithm development with little interest in immunology, (b) applied AI researchers who aim to integrate AI into biomedical sciences but often lack deep immunology knowledge, and (c) computational biologists who specialize in translating immunological data into AI models. The latter group is best suited to drive forward AI-driven immunological research. Regardless of their expertise, AI researchers must dedicate significant time to understanding immunological mechanisms to create meaningful models. The complexity of the immune system presents challenges not only for AI researchers but also for immunologists themselves. Even with advances in experimental techniques, immunologists frequently encounter unexpected immune behaviors that require continuous reevaluation of existing theories. AI can aid in identifying patterns and refining immunological hypotheses, but collaboration with experimental immunologists is essential to validate AI-generated insights. To bridge this gap, interdisciplinary teams should comprise immunologists, computational biologists, and AI researchers who collaborate to develop AI-driven models of immunology. Ultimately, AI-driven immunology holds immense potential; however, its success depends on interdisciplinary collaboration, careful validation, and the continuous refinement of computational models. By integrating AI into immunology, researchers can accelerate discoveries, enhance our understanding of immune responses, and improve immunotherapeutic strategies and patient outcomes.
